# Fluorescent cAMP Analogs: Enzymatic Synthesis, Unexpected Photophysical Behavior and Protein Kinase A Recognition

**DOI:** 10.1002/anie.6334998

**Published:** 2026-08-07

**Authors:** Deyuan Cong, Victor Dubois, Jamie Y. Lam, Yitzhak Tor

**Affiliations:** ^1^ Department of Chemistry University of California San Diego La Jolla California USA

**Keywords:** cyclic adenosine monophosphate, emissive nucleosides, enzyme kinetics, fluorescence, protein kinase A

## Abstract

Cyclic adenosine monophosphate (cAMP) is a second messenger, regulating critical physiological processes. We report the enzymatic synthesis, unique photophysical features, and biological activity of novel emissive cAMP analogs (c^th^AMP and c^tz^AMP), comprised of thiopheno‐ and isothiazolo‐based families of isomorphic ribonucleotides. The unexpectedly high brightness of c^th^AMP compared to ^th^ATP and ^th^AMP, its precursor and hydrolysis products, respectively, driven by an *anti*‐to‐*syn* conformational shift upon cyclization, is employed for real‐time monitoring of the adenylyl cyclase (AC) mediated synthesis and phosphodiesterase (PDE) mediated hydrolysis. Importantly, these emissive analogs effectively activate protein kinase A (PKA). This study provides critical insights into the biomolecular recognition and catalytic mechanism of cAMP‐regulating enzymes, establishing these isomorphic analogs as potential tools for investigating cAMP‐dependent protein–ligand interaction through fluorescence spectroscopy.

## Graphical abstract

**Cyclic adenosine monophosphate (cAMP) is a key cellular second messenger**. Adenylyl cyclase converts emissive isomorphic nucleoside triphosphates into fluorescent cAMPs. c^th^AMP displays unusually high brightness compared to ^th^ATP, its precursor, enabling real‐time monitoring of enzymatic reactions. These cAMP analogs effectively activate protein kinase A and are promising tools for investigating cAMP‐dependent protein–ligand interactions.

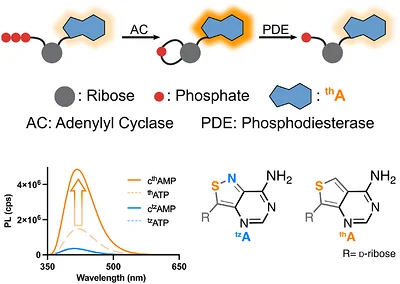

## 1 Introduction

Cyclic adenosine monophosphate (cAMP) serves as a pivotal second messenger in numerous intracellular signaling pathways, governing physiological processes that range from metabolic regulation to gene expression [1, 2]. Such signal transduction events rely on its specific binding to and activation of downstream proteins, most notably protein kinase A (PKA), cyclic nucleotide‐gated ion channels (CNGs), and the exchange protein directly activated by cAMP (EPAC) [3, 4, 5, 6, 7]. To elucidate the structural and functional dynamics of these interactions, various cAMP analogs have been employed, including derivatives designed to enhance membrane permeability, hydrolytic stability, or binding affinity [8, 9]. For instance, c(7‐deaza‐)AMP, in which the N7 nitrogen atom is replaced by C–H, exhibits increased lipophilicity and altered hydrogen‐bonding features. This modification often confers higher binding affinity to certain targets due to favorable hydrophobic interactions within the binding pocket [10, 11]. Additionally, fluorescent analogs, such as 2’‐*O*‐*N*‐methylanthraniloyl‐cAMP (MANT‐cAMP), 8‐fluo‐cAMP and cyclic ethenoAMP have been developed [12, 13, 14, 15]. These probes often possess significantly modified skeletons or carry bulky fluorophores, which can compromise critical recognition features and hence their applications [12, 13, 14, 15].

To address the challenge of visualizing nucleic acid recognition and dynamics while minimizing perturbations, our laboratory has established two families of isomorphic emissive nucleosides: the thiopheno‐ and isothiazolo‐based alphabets (denoted ^th^N and ^tz^N, respectively; see Figure 1) [16, 17]. These analogs were designed to be structurally isomorphic to their natural counterparts while possessing distinct and responsive photophysical features. They have been successfully deployed to probe the mechanisms of catalytic RNAs [18, 19], nucleoside‐metabolizing enzymes [20, 21, 22], cyclic dinucleotides [23], among other applications [24, 25, 26]. Notably, previous studies have demonstrated that ^tz^A and ^th^A can perform as 5’ initiators to be recognized by RNA polymerases for single incorporation into RNA transcripts via in vitro transcription [27]. Furthermore, their triphosphate derivatives serve as substrates for nicotinamide mononucleotide adenylyltransferase 1 (NMNAT1), enabling the enzymatic synthesis of fluorescent NAD^+^ analogs [28, 29, 30].

**FIGURE 1:**
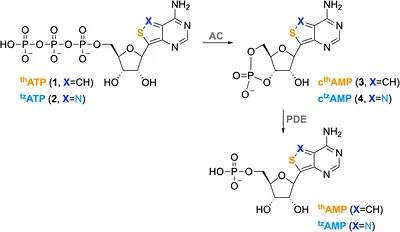
AC converts ^th/tz^ATP to the corresponding emissive c^th/tz^AMP analogs, which can be subsequently hydrolyzed to the corresponding emissive ^th/tz^AMP analogs by PDE.

We hypothesized that ^th^A and ^tz^A, as emissive adenosine analogs, could be exploited for the investigation of enzymes associated with cellular cAMP synthesis (e.g., cAMP adenylyl cyclase; AC) and degradation (e.g., phosphodiesterase, PDE). Herein, we disclose the enzymatic synthesis of these cAMP analogs and show that c^th^AMP is much brighter than its triphosphate precursor. Notably, variable temperature NMR analyses and photophysical characterizations, alongside computational calculations, reveal that this fluorescence enhancement is driven by an *anti*‐to‐*syn* conformational shift. The significant fluorescent enhancement seen upon c^th^AMP formation is utilized to monitor AC‐mediated phosphodiester formation and PDE‐induced hydrolysis in real‐time. Furthermore, we reveal that these emissive cAMP analogs effectively activate PKA. The resulting structure‐activity relationship provides insight into the substrate recognition and catalytic mechanisms of both AC and PKA, establishing these isomorphic analogs as potential tools for investigating cAMP‐dependent protein–ligand interaction using fluorescence‐based techniques.

## 2 Results and Discussion

### 2.1 Enzymatic Synthesis of cAMP Analogs

ATP is the substrate utilized by AC for cAMP synthesis. The corresponding emissive triphosphate analogs (^th^ATP and ^tz^ATP, **1** and **2,** respectively, Figure 1) were chemically synthesized from their nucleoside precursors according to previously reported protocols [29, 30]. Treatment with AC (Recombinant *B. pertussis*) in the presence of calmodulin facilitates enzymatic 3’,5’‐phosphodiester formation (Figure 1). HPLC and mass spectrometry analyses showed that 0.15 mM of ATP and ^tz^ATP, subjected to 1.08 µg/mL AC and 0.54 µg/mL calmodulin at 30°C, underwent complete conversion into the corresponding cAMP products within 5 and 20 min, respectively (Figure 2). The thienopyrimidine analog ^th^ATP (**1**, Figure 1) displayed slower conversion, reaching 90% completion over a longer (60 min) incubation period (Figure 2).

**FIGURE 2:**
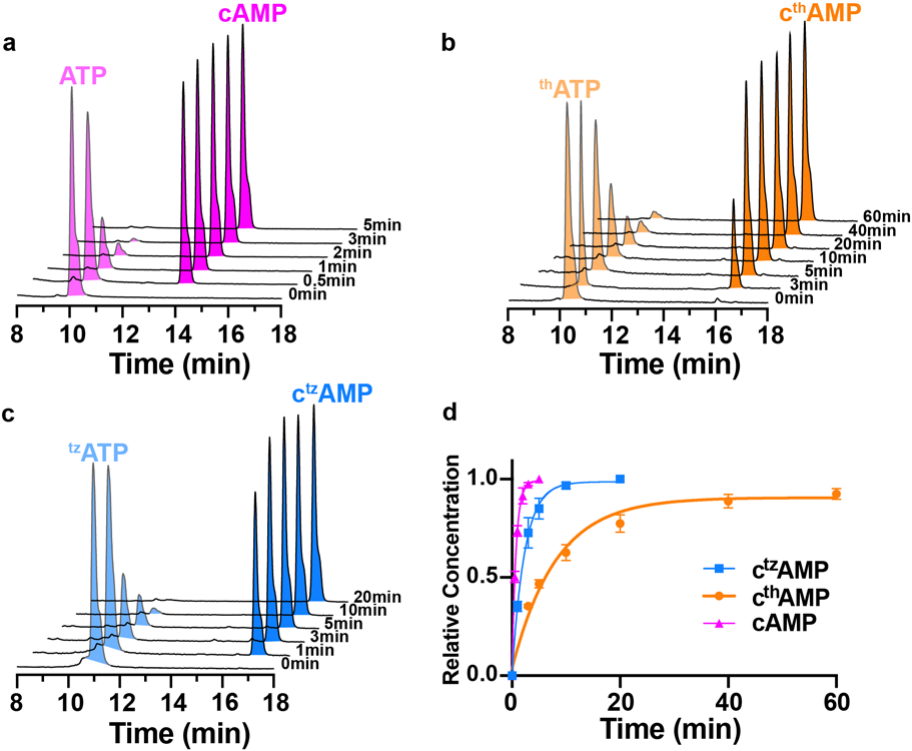
AC‐mediated enzymatic synthesis of cAMP and its analogs c^th^AMP, c^tz^AMP. (a) UV‐monitored HPLC chromatograms (260 nm) of the AC‐mediated synthesis of cAMP. (b) UV‐monitored HPLC chromatograms (341 nm) of the AC‐mediated synthesis of c^th^AMP. (c) UV‐monitored HPLC chromatograms (338 nm) of the AC‐mediated synthesis of c^tz^AMP. (d) Normalized time courses of AC‐mediated synthesis of cAMP, c^th^AMP and c^tz^AMP. Reactions were duplicated. Error bars indicate standard deviation (SD).

To further evaluate the enzyme's competitive substrate preferences, an equimolar mixture of ATP, ^tz^ATP, and ^th^ATP (0.2 mM mixture, 1:1:1 ratio) was incubated with AC (1.08 µg/mL) and calmodulin (0.54 µg/mL) at 30°C. The enzyme produced all three corresponding products, cAMP, c^tz^AMP, and c^th^AMP, displaying the highest catalytic efficiency with ATP, followed by ^tz^ATP, and finally ^th^ATP (Figure S1), as observed for the individual reactions. Although differing in rates, these observations illustrate AC's tolerance to ^tz^ATP and ^th^ATP, our emissive ATP analogs, and provide a simple enzymatic pathway for synthesizing both cAMP analogs.

### 2.2 Photophysical Features of c^tz^AMP and c^th^AMP

To assess the basic photophysical features of c^tz^AMP and c^th^AMP, their steady‐state absorption and emission spectra were recorded and compared to the corresponding adenosine analogs (Table 1 and Figure 3). In comparison to ^tz^ATP and ^th^ATP, their precursors, c^tz^AMP and c^th^AMP feature the same absorption maxima at λ_abs_ = 338 and 341 nm, respectively. When comparing the emission quantum yields (*Φ_f_*) of the fluorescent cAMP analogs to those of the precursor triphosphates, c^tz^AMP and ^tz^ATP display practically the same value (*Φ_f_ =* 0.051 and 0.053, respectively) as expected. Surprisingly, however, c^th^AMP is unusually bright, displaying a much higher emission quantum yield compared to ^th^ATP, its precursor (*Φ_f_ =* 0.67 vs. 0.21, respectively).

**TABLE 1:** Photophysical properties of emissive nucleotides and cAMP analogs.

	*λ*_abs_ [nm]	*ϵ* [m ^−1^ cm^−1^]	*λ*_em_ [nm]	*Φ_f_* ^c^	τ*_f_* ^d^ [ns]
^th^ATP^a^	341	7.4 × 10^3^	420	0.21	2.90
c^th^AMP^b^	341	7.4 × 10^3^	420	0.67 ± 0.03	9.93
^th^AMP^b^	341	7.4 × 10^3^	420	0.19 ± 0.01	2.63
^tz^ATP^a^	338	7.8 × 10^3^	410	0.053	0.70
c^tz^AMP^b^	338	7.8 × 10^3^	410	0.051 ± 0.002	0.69
^tz^AMP^b^	338	7.8 × 10^3^	410	0.040 ± 0.002	0.52

^a^ Previously reported values, see corresponding Ref. 16 and 17.

^b^ The ϵ value was assumed to be unaffected by the presence of cyclic phosphodiester or monophosphate. Quantum yield values (*Φ_f_*) are presented as mean ±  standard deviation (SD). Measurements were performed in 1 × PBS buffer (without Mg^2+^, Ca^2+^).

^c^ Fluorescence quantum yield, standard: quinine bisulfate (QBS) in 0.5 M H_2_SO_4_ (*Φ_f_ =* 0.546).

^d^ Fluorescence lifetimes were calculated according to the Strickler–Berg equation [31, 32].

**FIGURE 3:**
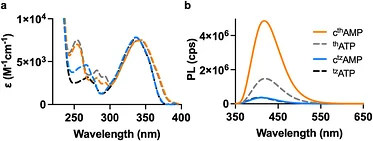
Absorption (a) and emission (b) spectra of c^th^AMP (orange), ^th^ATP (grey), c^tz^AMP (blue), and ^tz^ATP (black) in 1 × PBS buffer (without Mg^2+^, Ca^2+^). The emission spectra were normalized to an optical density of 0.1 at the excitation wavelengths (341 and 338 nm for derivatives containing ^th^A and ^tz^A, respectively).

A priori, the dramatic fluorescence enhancement seen upon converting ^th^ATP to c^th^AMP is surprising. The fluorescence efficiency of a fluorophore relies exclusively on the competition between its radiative (*k*_r_) and nonradiative (*k*_nr_) rate constants. Since the former is governed solely by the transition probability between the ground and excited states, such fluorescence enhancement is unlikely to arise from *k*_r_, as the emitting chromophore remains intact. To support this hypothesis, fluorescence lifetimes were calculated using the Strickler–Berg equation [31, 32], thereby allowing us to extract *k*_r_ and *k*_nr_ constants. Although *k*_r_ remains in the same order of magnitude for both ^th^ATP (7.25 × 10^7^ s^−1^) and c^th^AMP (6.75 × 10^7^ s^−1^), *k*_nr_ has been dramatically altered by the modification of the fluorophore's microenvironment upon the intramolecular formation of the 3’,5’‐phosphodiester bond (2.73 × 10^8^ s^−1^ and 3.32 × 10^7^ s^−1^ for ^th^ATP and c^th^AMP, respectively, Table S1).

This 3′,5′‐phosphodiester cyclization is known to affect the sugar pucker, thereby lowering the energy barrier between the *syn* and *anti* conformations. In fact, native cAMP has been shown to populate both conformers in solution and in the solid state (Figure S2) [33]. Given the isomorphicity of our emissive analogs [16, 17], and the common non‐radiative relaxation pathways, we hypothesize that populating the *syn* conformation could be the primary source of this unprecedented fluorescence enhancement. Indeed, among non‐radiative processes, solvent‐induced relaxation plays a central role, particularly in water, in which H‐bonding frequently diminishes fluorescence [34]. Thereby, cloaking one H‐bond acceptor in a *syn* conformation (i.e., the “N3”, Figure 4a), thus diminishing water accessibility, should contribute to reducing *k*_nr_. To experimentally assess this hypothesis, solvent isotope effects were evaluated in methanol (CH_3_OH) and deuterated methanol (CD_3_OD). A noticeable fluorescence enhancement was observed for ^th^A and ^th^ATP in a deuterated solvent (ca. two‐fold), whereas the conformationally constrained c^th^AMP remained insensitive (Figure S3 and Table S2).

**FIGURE 4:**
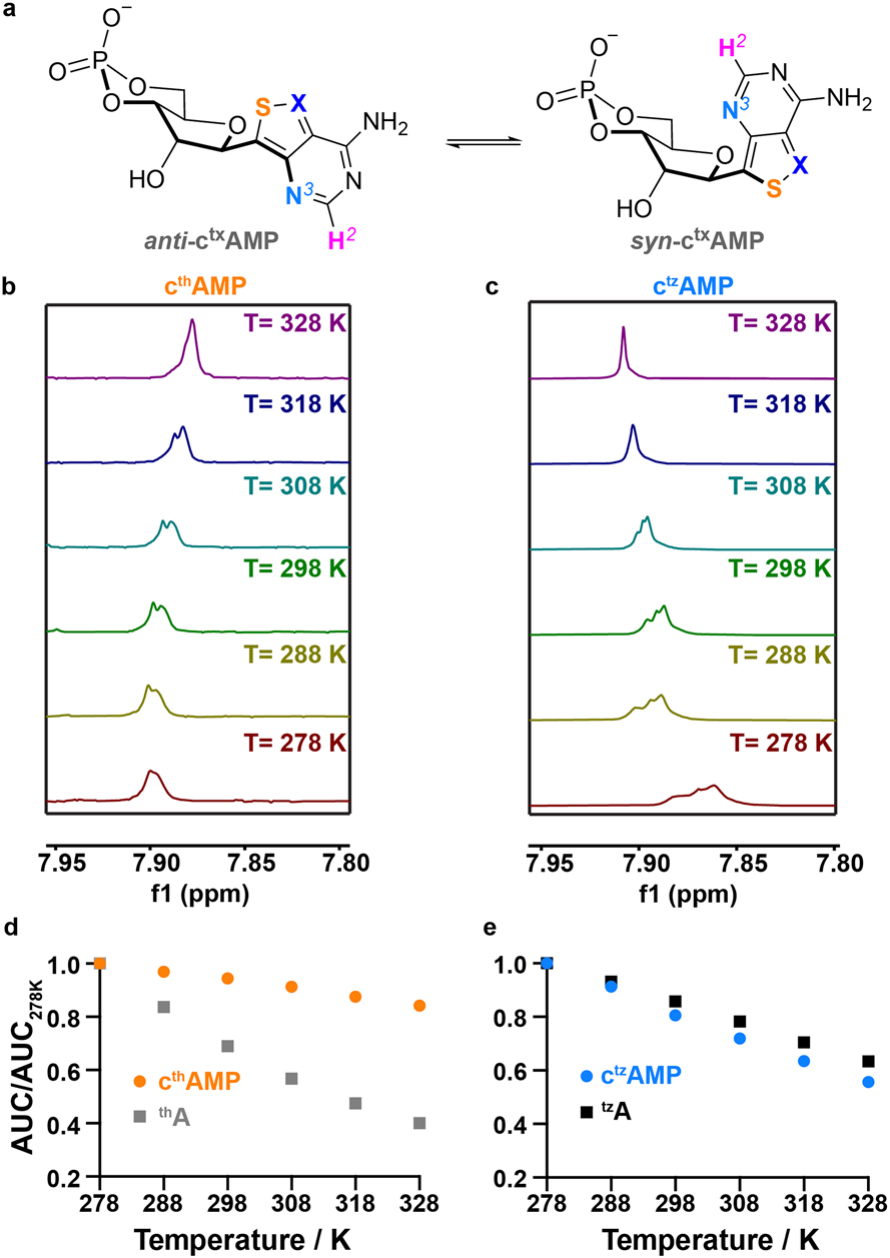
Temperature‐dependent ^1^H‐NMR and photophysical characterization of cAMP analogs. (a) *Anti* and *syn* conformations of the emissive cAMP analogs. (b, c) Variable‐temperature ^1^H‐NMR, showing the pyrimidine H2 of c^th^AMP (left) and c^tz^AMP (right). (d, e) Temperature‐dependent relative fluorescence intensity of ^th^A and c^th^AMP (left), as well as ^tz^A and c^tz^AMP (right). Relative fluorescence intensities were obtained by integrating the area under the emission curve (AUC) and normalizing to the initial values at 278 K (AUC_278K_). Measurements were done in triplicate. Error bars, indicating standard deviation (SD), are small and have been removed for clarity. See Supporting Information for an alternative depiction including the error bars (Figure S4).

Although the non‐chromophoric backbone is often overlooked when investigating nucleosides’ photophysics, even such subtle modifications can deeply alter their dynamics and thereby their photophysical properties [35]. To investigate the hypothesized *syn*/*anti* conformational dynamics, we performed both temperature‐dependent ^1^H NMR and fluorescence spectroscopy experiments.

While ^1^H‐NMR spectra of the nucleoside precursors (^th^A and ^tz^A) display single distinct peaks [16, 17], spectra taken for both c^th^AMP and c^tz^AMP show multiple overlapping signals for the aromatic H2 proton at ambient temperatures (ca. 298 K). This behavior is consistent with the distinct chemical shifts reported for *syn* and *anti* conformations in various purine analogs [36, 37]. Furthermore, the existence of a dynamic *anti*‐to‐*syn* equilibrium in native cAMP has been established by temperature‐dependent NMR relaxation studies [38]. To characterize this behavior in our fluorescent analogs, ^1^H‐NMR spectra were recorded across a temperature range of 278 to 328 K. These spectra show further broadening at low temperatures and coalescence into a sharper dominant peak at elevated temperatures (Figure 4b,c), thus suggesting conformational dynamics. Coalescence temperatures of approximately 320 and 310 K were determined for c^th^AMP and c^tz^AMP, respectively, suggesting a rather small energy barrier.

To assess the impact of such conformational dynamics on the photophysical properties of c^tz^AMP and c^th^AMP, absorption and steady‐state emission spectra were recorded within the same temperature range used above. Relative fluorescence intensities were obtained by integrating the emission curves and normalizing the values to the intensity at 278 K (AUC/AUC_278K_). As expected, all four emissive species (^tz^A, ^th^A, c^tz^AMP, c^th^AMP) showed a decrease in fluorescence intensity with increasing temperature (Figures 4d,e and S5). A disparity emerged, however, when comparing the thieno‐ and isothiazolo‐ derivatives. While the relative fluorescence values (AUC_328K_/AUC_278K_) of ^tz^A (0.63) and c^tz^AMP (0.56) displayed similar temperature dependency (slope) and overall relative drop, a significant difference was observed between ^th^A (0.40) and c^th^AMP (0.84) (Figure 4d). Notably, despite the inherent fluorescence loss typically induced by elevating the temperature, the emission of c^th^AMP remains relatively steady even as rising temperature facilitates a fast equilibrium between the *syn* and *anti* conformations. This evidence supports the notion that the fluorescence enhancement is dependent on the prevalence of the *syn* conformation.

Why does the 3’,5’‐cyclic scaffold trigger fluorescent enhancement in the thieno‐analog (c^th^AMP) and not for its isothiazolo counterpart (c^tz^AMP)? In c^th^AMP, the hydrophobic shielding in the *syn* conformation clearly attenuates solvent‐induced non‐radiative deactivation pathways, thereby augmenting the emission quantum yield. In contrast, the presence of the basic “N7” in the isothiazolo[4,3‐*d*]pyrimidine scaffold (e.g., **2** and **4**) provides an additional H‐bond accepting site, which is absent in the thieno[3,4‐*d*]pyrimidine scaffold (Figure 4a). This inherently increases *k*_nr_ in protic solvents for ^tz^N‐containing derivatives (Tables 1 and S1). The observation that 3’,5’‐cyclic phosphodiester formation doesn't impact the fluorescence properties of the conformationally dynamic c^tz^AMP compared to those of ^tz^A, despite the accessibility of the “N3” shielded *syn* conformation, suggests that, independently of solvent‐induced relaxation, c^th^AMP and c^tz^AMP likely adopt two distinct non‐radiative decay pathways. Solvent isotope effects helped corroborate this assertion. Unlike the ^th^A nucleoside family, emission spectra taken in CH_3_OH and CD_3_OD revealed that all derivatives within the ^tz^A family (^tz^A, ^tz^ATP and c^tz^AMP) exhibited insensitivity to deuteration (Figure S3 and Table S2).

To further support our interpretation and gain further insight into the conformational dynamics of the newly synthesized cAMP surrogates, quantum‐chemical calculations were performed using Gaussian 9 [39] along with GaussView 6 [40], and Multiwfn [41, 42]. In addition to c^th^AMP and c^tz^AMP, ^th^AMP and ^tz^AMP were considered to closely mimic ^th^ATP and ^tz^ATP (while reducing the number of atoms). In line with previous computational investigations on thieno‐ and isothiazolo‐nucleoside analogs [43, 44, 45, 46, 47], ground‐ and excited‐state geometries of their *syn* and *anti* conformations were first optimized at the (TD)DFT level of theory using B3LYP/6‐31+G(d,p) [48, 49, 50]. The polarizable continuum model (PCM) [51, 52] was employed to account for implicit solvation in water. The vertical energy transitions were computed using the well‐established corrected linear response (cLR) [53]. Comparison with the experimental data (Table 2) suggests that the combination of B3LYP and 6–31+G(d,p) gives a realistic description of the excited‐state dynamics of our derivatives in aqueous media (Table 2). Interestingly, the analysis of the oscillator strengths (Table 2) reveals similar elevated values for both ^th^AMP and c^th^AMP in their *syn* and *anti* conformations. This confirms that the fluorescence enhancement observed for c^th^AMP arises from a *k*_nr_ reduction upon cloaking “N3” rather than an increase of the transition probability (*k*_r_). For ^tz^AMP and c^tz^AMP, however, cloaking the “N3” is not sufficient, as the *syn* conformations display a much lower transition probability than the *anti* due to a mixing of n‐π* and π‐π* transitions with a non‐negligible amount of n‐π* character. This is consistent with previous studies on ^tz^A, showing the close proximity of π‐π* and n‐π* states [46, 54].

**TABLE 2:** Comparison of the experimental data (in DPBS) and the computed vertical energy transitions associated with their oscillator strengths (*f*_01_ for the excitation and *f*_10_ for the emission) using B3LYP/6‐31+G(d,p) IEFPCM water.

Compound	λ_abs_^exp^ [nm]	λ_abs_^theo^ [nm]	ε [M^−1^cm^−1^]	*f_01_*	λ_em_^exp^ [nm]	λ_em_^theo^ [nm]	Φ*_f_*	*f_10_*
**^th^AMP (syn)**	341	335	7.4 × 10^3^	0.1841	420	418	0.21	0.2851
**^th^AMP (anti)**		339		0.1640		427		0.2535
**c^th^AMP (syn)**	341	335	7.4 × 10^3^	0.1905	420	417	0.67 ± 0.03	0.2987
**c^th^AMP (anti)**		338		0.1821		427		0.2704
**^tz^AMP (syn)**	338	338	7.8 × 10^3^	0.1691	410	458	0.053	0.0617
**^tz^AMP (anti)**		333		0.1881		421		0.2555
**c^tz^AMP (syn)**	338	339	7.8 × 10^3^	0.1868	410	449	0.051 ± 0.002	0.0806
**c^tz^AMP (anti)**		335		0.1913		423		0.2920

Finally, we performed potential energy surface (PES) scans around the glycosidic bond at the ground and excited states (Figure 5). Not surprisingly, a higher proportion of the *syn* conformation was found in c^th^AMP and c^tz^AMP due to the alteration of the sugar pucker. The ratios of the *syn* and *anti* conformations at the ground state, 40/60 and 15/85 for c^th^AMP (Figure 5b) and c^tz^AMP (Figure 5d), respectively, corroborate the VT ^1^H NMR experiment. Interestingly, the energy barriers between these two species also correspond to the coalescence temperatures, which follow the same trend. The differences between the thieno‐ and the isothiazolo‐ derivatives are, however, much more pronounced in the excited state. Although it is counter‐productive due to the presence of the dark n‐π* state, the proportions of the *syn* conformer increase surprisingly in the excited state for the isothiazolo[4,3‐*d*]pyrimidine derivatives. On the contrary, the thieno[3,4‐*d*]pyrimidine derivatives follow the opposite trend. Nonetheless, in addition to the high proportion of *syn* observed in the ground state, we found that the proportion of the *syn* conformation of c^th^AMP (10%) was 2.5 times that of ^th^AMP (4%) in the excited state, which appears consistent with the threefold enhancement to the fluorescence quantum yield (Figure 5a,b).

**FIGURE 5:**
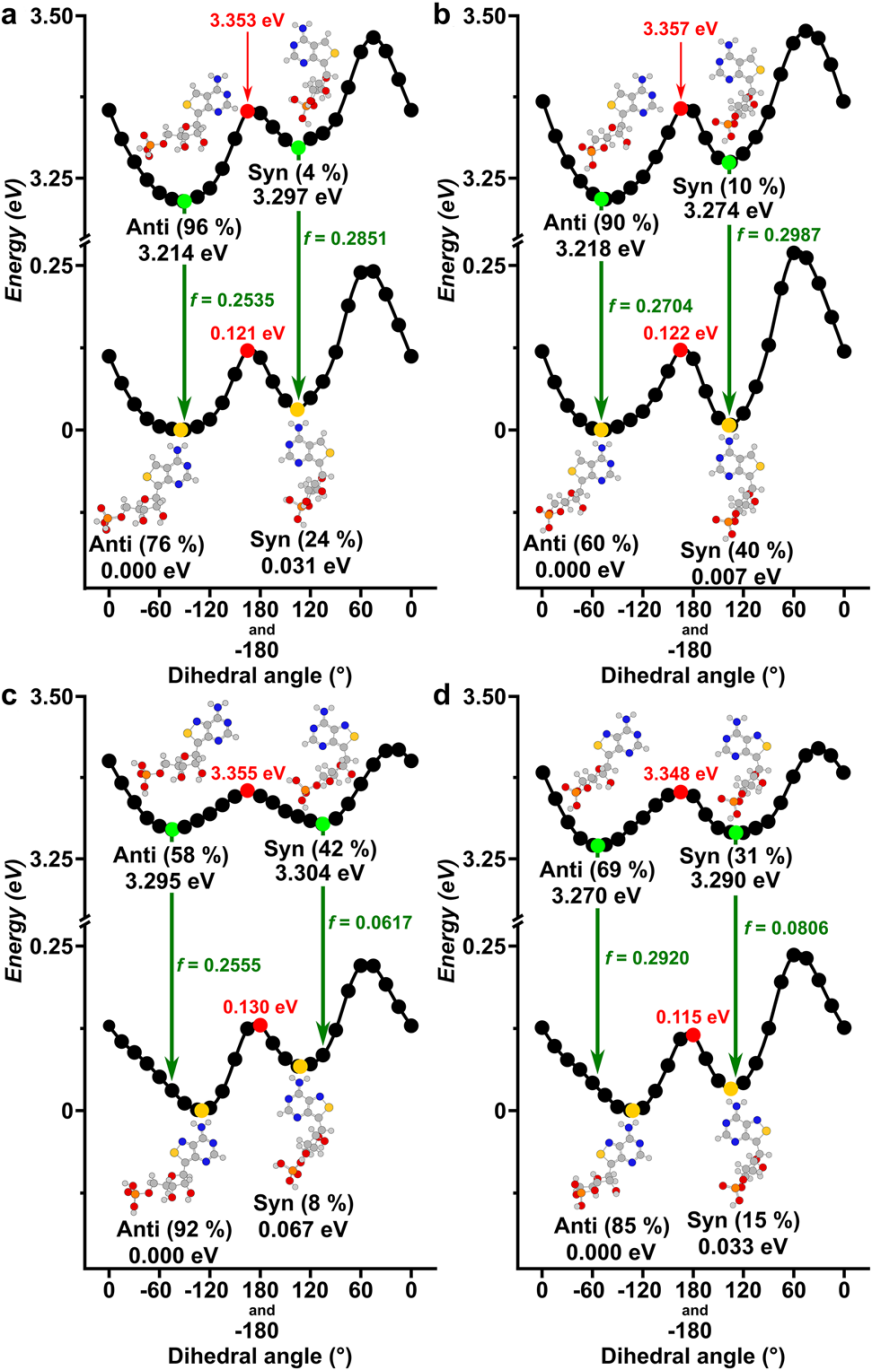
Ground and excited‐state potential energy surface scans around the glycosidic bond for (a) ^th^AMP, (b) c^th^AMP, (c) ^tz^AMP, and (d) c^tz^AMP using B3LYP/6‐31+G(d,p) IEFPCM Water.

Taken together, our photophysical investigations combined with TD‐DFT studies clearly demonstrate that the ability of c^th^AMP to adopt a *syn* conformation induces a major change in its fluorescence efficiency compared to the linear phosphate derivatives. While evaluating alternative analogs, like the isomeric 2’,3’‐cAMP, which predominantly favors the *anti*‐conformation (Figure S6), may provide a valuable comparison, the unique photophysical features of c^th^AMP can be exploited to monitor, in real time, the enzymatic activity of AC and PDE as discussed below.

### 2.3 Monitoring AC‐Mediated Synthesis and PDE‐Mediated Cleavage of c^th^AMP

The dramatic differences in emission quantum yield between ^th^A (or ^th^ATP) and c^th^AMP can be exploited for monitoring AC‐mediated enzymatic conversions in real‐time. Following the nucleotides’ emission, incubation of ^th^ATP with AC under the same conditions as the HPLC‐monitored reactions indeed shows a significant fluorescence enhancement as the synthesis progresses (Figure 6a). To compare the chromatographic and spectroscopic methods, the integrated area of each HPLC c^th^AMP product peak and each emissive spectrum data set were normalized and graphically depicted (Figure 6b). The apparent kinetic rate constants, obtained by fitting pseudo‐first‐order curves, yielded *k_app_* values of 0.12 and 0.09 min^−1^ for HPLC‐ and fluorescence‐monitored reactions, respectively. Notably, the apparent reaction rate generated by fluorescence is in reasonable agreement with the value extracted from the HPLC‐analyzed reactions. The minor deviation observed (Figure 6b) likely results from their different execution protocols: HPLC requires the reaction to be quenched at each designated time point, while real‐time fluorescence monitors ongoing reactions where all species are present. Consequently, the fluorescence data reflect the overall progress during the scan rather than a single frozen time point. Both techniques reflect the same molecular event, thus suggesting that fluorescence can facilitate real‐time monitoring of AC‐mediated c^th^AMP synthesis.

**FIGURE 6:**
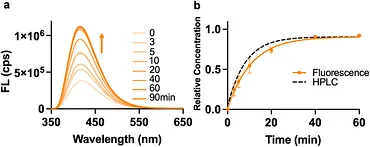
(a) AC‐mediated cyclization monitored by emission spectra for c^th^AMP (excitation at 341 nm). (b) AC‐mediated synthesis as a function of time, monitored by HPLC (black, dashed line) and fluorescence (orange, solid line). Assay was performed in triplicate. Error bars indicate standard deviation (SD).

To provide further support for the formation of the cyclic phosphodiester as the primary factor leading to the fluorescence enhancement observed, PDE1C (Human recombinant full‐length), a cA/GMP‐specific phosphodiesterase that cleaves the cyclic phosphodiester to the corresponding monophosphate, was added to the AC‐mediated synthesis cuvette when the reaction plateaued. This hydrolysis process was monitored by steady‐state fluorescence spectroscopy in real‐time. A continuous decrease in fluorescence intensity was observed, with an endpoint comparable to that of the AC‐mediated synthesis at time 0 (Figure 7b). This is consistent with the fact that ^th^AMP, the hydrolysis product, is as weakly fluorescent as ^th^ATP (Table 1 and Figure S7). This further supports the notion that the formation of c^th^AMP, capable of populating the *syn* conformation, is the predominant cause of the observed fluorescence enhancement.

**FIGURE 7:**
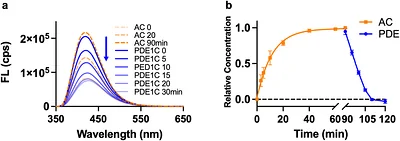
(a) AC‐mediated cyclization and PDE1C‐mediated hydrolysis of c^th^AMP monitored by emission spectra (excitation at 341 nm). (b) Time dependence of AC‐mediated synthesis and PDE1C‐mediated hydrolysis. Assay was done in triplicate. Error bars indicate standard deviation (SD).

### 2.4 Activation of cAMP‐Dependent PKA by Emissive cAMP Analogs

An important target of cAMP in eukaryotic cells is the cAMP‐dependent PKA, which regulates cell division, metabolism, and apoptosis [55]. The heterotetrameric holoenzyme is composed of two regulatory and two catalytic subunits [56], and remains inactive in the absence of cAMP. As the cellular concentration of cAMP increases, binding to the regulatory subunits releases the catalytic subunits, which mediate protein phosphorylation of diverse protein targets [57]. We tested the effect of our synthesized emissive cAMP analogs on PKA activation using a cAMP‐Glo luminescence‐based coupled assay (Promega, V1501), and compared them to cAMP and the enzymatically synthesized c(7‐deaza‐)AMP. Sigmoidal concentration‐response fitting of the resulting data sets yielded half‐maximum activation values (*EC*_50_) of 13.7 ± 1.1, 19.5 ± 3.8, 26.4 ± 4.2, and 54.3 ± 1.9 nM for cAMP, c(7‐deaza‐)AMP, c^th^AMP, and c^tz^AMP, respectively (Figure 8). c(7‐deaza‐)AMP showed the highest PKA activation potency among the three modified cAMP analogs. Intriguingly, c^th^AMP, which is considered to be less isomorphic than c^tz^AMP [17], showed higher potency, illustrating the context dependency of any nucleoside isostere. Considering the structures of the three cAMP analogs examined, both the c(7‐deaza‐)AMP and c^th^AMP lack the purines’ N7. This suggests that binding to the regulatory subunit of PKA might not be N7‐dependent, and that ligand hydrophobicity could be a factor influencing PKA activation. This is supported by the crystal structures of cAMP‐bound PKA complexes (Figure S8), which reveal that the purine nucleobase predominantly engages in aromatic–aromatic interactions with neighboring Trp and Tyr residues within the protein's hydrophobic pocket. Consequently, increased hydrophobicity and desolvation of the analogs could strengthen these aromatic interactions, thereby increasing activation potency [58, 59].

**FIGURE 8:**
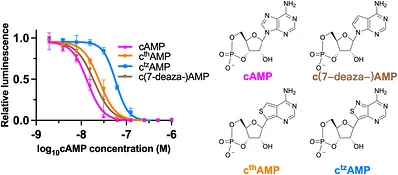
Effect of cAMP and its analogs on activation of PKA. Increasing the concentration of cAMP and its analogs leads to enhanced PKA activity, which is inversely proportional to the luminescence observed, as measured by a luciferase/luciferin coupled assay (cAMP‐Glo Assay, Promega, V1501; see Supporting Information, Section 3).

## 3 Conclusion

An efficient enzymatic pathway for the synthesis of emissive cyclic AMP analogs, c^tz^AMP and c^th^AMP, has been demonstrated. AC exhibits tolerance for nucleobase‐modified triphosphate precursors, leading to complete conversion to the corresponding cyclic phosphodiesters, albeit with varying catalytic efficiencies. To our surprise, c^th^AMP formation is associated with a remarkable fluorescence enhancement, with a quantum yield more than 3‐fold higher than that of ^th^ATP, its precursor. Temperature‐dependent NMR, fluorescence spectra, and TD‐DFT studies suggest that the formation of the cyclic phosphodiester, which augments the population of the *syn* conformer, alters the fluorophore's microenvironment and diminishes non‐radiative decay pathways. Bright c^th^AMP analogs thus serve as a powerful tool for real‐time monitoring of AC and PDE activities. While the latter can be subcellular compartment‐, cell‐, or tissue‐specific, structural analysis of various phosphodiesterases, such as PDE‐4 [60] and PDE‐10 [61], further suggests the potential compatibility of these analogs across different PDE families. Consequently, these analogs can provide new fluorescent probes for investigating cAMP‐dependent protein–ligand interactions through fluorescence spectroscopy.

## Author Contributions

**Deyuan Cong**: writing – original draft, investigation, methodology, validation, data curation. **Victor Dubois**: visualization, validation, investigation, writing – original draft, data curation, formal analysis, software, methodology. **Jamie Y. Lam**: methodology, investigation, software, data curation, validation. **Yitzhak Tor**: conceptualization, funding acquisition, writing – original draft, validation, writing – review and editing, project administration, supervision, resources, formal analysis, visualization.

## Conflicts of Interest

The authors declare no conflicts of interest.

## Supporting information




**Supporting File**: anie73935‐sup‐0001‐SuppMat.pdf.

## Data Availability

The data that support the findings of this study are available on request from the corresponding author. The data are not publicly available due to privacy or ethical restrictions.
