# Supplementary material for: Fluorescent cAMP Analogs: Enzymatic Synthesis, Unexpected Photophysical Behavior and Protein Kinase A Recognition
Source: Angew Chem Int Ed Engl. 2026 Aug 7;65(41):e6334998. doi: 10.1002/anie.6334998 (PMC13638118; doi:10.1002/anie.6334998)
Supplement: Supplementary file 1 [file ANIE-65-e6334998-s001.pdf]

Supporting Information

Fluorescent cAMP Analogs: Enzymatic Synthesis, Unexpected Photophysical Behavior and Protein Kinase A Recognition

Deyuan Cong, Victor Dubois, Jamie Y. Lam and Yitzhak Tor*

Supplementary Information

1. Materials and Methods *1*

2. Synthetic Procedures *1*

3. Enzymatic experiments *1*

4. Photophysical characterization *3*

5. Supporting Figures *5*

5.1 AC-mediated Synthesis of ATPs Mixture 5

5.2 Crystal Structures of 3',5'-cAMP 6

5.3 Solvent Isotope Effect of Quantum Yield in thA and ^{tz}A Families 6

5.4 Emissive Spectra upon Variable Temperatures of cAMP Analogs and Their Corresponding Nucleosides 7

5.5 Crystal Structures of 2',3'-cAMP 9

5.6 Emission spectra of thAMP and ^{tz}AMP in PBS buffer 9

5.7 X-ray Crystal Structures of PKA-bound cAMP 9

5.8 HPLC Traces of PDE-mediated Hydrolysis 10

5.9 Emission Spectra upon Variable Structures of thA and ^{tz}A Families in CH_3OH and CD_3OD 10

5.10 NMR Spectra of Fluorescent cAMP Analogs 12

6. Supporting Tables *19*

7. Computational Analysis of cAMP Analogs *19*

7.1 Geometry Optimization 19

7.2 Frontier Molecular Orbitals 28

7.3 Vertical Transition Energies 29

7.4 Potential Energy Surfaces in Water 29

References *30*

1. Materials and Methods

Reagents, buffers and salts were purchased from Sigma-Aldrich, Fluka, TCI, Acros and Synchem, Inc. (Elk Grove, IL), and were used without further purification unless otherwise specified. NMR solvents were purchased from Cambridge Isotope Laboratories (Andover, MA). All reactions were monitored with analytical TLC (Merck Kieselgel 60 F_{254}). All experiments involving air and/or moisture sensitive compounds were carried out under an argon atmosphere. Column chromatography was carried out with silica gel particle size 40–63 μm. NMR spectra were obtained on a Jeol 400 MHz spectrometer and a Bruker 600 MHz spectrometer at the NMR Facility at the UCSD Chemistry and Biochemistry Department. ESI-TOF mass spectra were obtained on an Agilent 6230 HR-ESI-TOF MS at the Molecular Mass Spectrometry Facility at the UCSD Chemistry and Biochemistry Department.

2. Synthetic Procedures

thATP and ^{tz}ATP were chemically synthesized according to previously reported procedures.[1, 2]

For preparative-scale enzymatic synthesis of cAMP analogs, the adenosine 5'-triphosphate analogs (1 mM) were incubated at 30 ℃ with 3 μg/mL adenylate cyclase (AC; Recombinant *B. pertussis*; R&D Systems, Catalog 8270-AC) and 1.5 μg/mL calmodulin (Sigma, Catalog P1431) in a reaction buffer containing 25 mM Tris, pH 7.0, 10 mM $MgCl_2$ and 50 μM $CaCl_2$. After incubation for 6 hours, the reaction mixture was filtered through a 0.22 μm filter. The supernatant was subsequently purified by Sepax Bio C-18 column (4.6 × 260 mm, 5 μm particle size) with a gradient of 0.5%–20% of 50 mM TEAA (Triethylammonium acetate, pH 7.5) in ACN in 20 min at 25 ℃ on an Agilent 1200 series HPLC system (Agilent Technologies). Collected HPLC fractions were lyophilized with Labconco FreeZone 2.5 lyophilizer and re-dissolved in autoclaved water. UV spectroscopy was used to determine the concentration of each solution with the following extinction coefficients: 7790 L mol^{-1} cm^{-1} for c^{tz}AMP (338 nm), 7440 L mol^{-1} cm^{-1} for cthAMP (341 nm) and 9500 L mol^{-1} cm^{-1} for c(7-deaza-)AMP (271 nm). Characterizations of c^{tz}AMP: ^{1}H NMR (400 MHz, D_2O) δ 8.08 (s, 1H), 5.68 (s, 1H), 4.47–4.37 (m, 2H), 4.26–4.17 (m, 3H). ^{31}P NMR (162 MHz, D_2O) δ –1.59 (indirect reference to ^{1}H NMR via IUPAC Xi(Ξ_x) ratio).[3] ESI-HRMS calculated for $[C_{10}H_{10}N_4O_6PS]^-$ 345.0064, found 345.0062. Characterizations of cthAMP: ^{1}H NMR (400 MHz, D_2O) δ 8.35 (s, 1H), 8.01 (s, 1H), 5.68 (s, 1H), 4.42–4.33 (m, 2H), 4.20–4.11 (m, 3H). ^{31}P NMR (162 MHz, D_2O) δ –6.02 (indirect reference to ^{1}H NMR via IUPAC Xi(Ξ_x) ratio).[3] ESI-HRMS calculated for $[C_{11}H_{11}N_3O_6PS]^-$ 344.0112, found 344.0115.

3. Enzymatic experiments

For kinetic studies of cAMP analog synthesis, the reaction was initiated by incubating the adenosine 5'-triphosphate analogs (0.15 mM) with 1.08 μg/mL adenylate cyclase (AC, Recombinant *B. pertussis*; R&D system, Catalog 8270-AC) and 0.54 μg/mL calmodulin (Sigma, Catalog P1431) in a buffer containing 25 mM Tris, pH 7.0, 10 mM $MgCl_2$ and 50 μM $CaCl_2$ at 30 ℃. Aliquots of the reaction mixture (9 μL) were taken

out at designated time points and were quenched with EDTA (20 μL 10 mM), and the solution (25 μL) was subjected to HPLC analysis after filtration. Separation was done on a Sepax Bio C-18 column (4.6 × 260 mm, 5 μm particle size) with a gradient of 0.5 – 20% of 50 mM TEAA, pH 7.5 in ACN in 20 min at 25 ℃ on an Agilent 1200 series HPLC system (Agilent Technologies).

For competitive AC-mediated synthesis of cAMP analogs, similar procedures were used as described above, but with a starting substrate of ATP, ^{tz}ATP and thATP mixture at a total concentration of 0.20 mM (1:1:1, concentration ratio, yielding 0.067 mM for each substrate).

AC-mediated synthesis and PDE-1C-mediated hydrolysis monitored with steady-state fluorescence spectroscopy. For AC-mediated synthesis, adenosine 5'-triphosphate analogs (0.15 mM) were incubated with 1.08 μg/mL adenylate cyclase (AC; Recombinant *B. pertussis*; R&D system, Catalog 8270-AC) and 0.54 μg/mL calmodulin (Sigma, Catalog P1431) in a buffer containing 25 mM Tris, pH 7.0, 10 mM $MgCl_2$ and 50 μM $CaCl_2$ at 30 ℃. The reactions were performed in a 125 μL cuvette. All emission spectra were collected from 350 nm to 650 nm at designated time points (excitation wavelength = 341 nm, Temperature = 30 ℃).

Once the emission intensity reached a plateau, PDE1C (Human recombinant full length; BPS Bioscience, Catalog 60013; 2 μL 0.14 mg/mL) was added directly to the same cuvette. The mixture was incubated at 30 ℃ and the emission spectra were collected at designated time intervals as above.

The reaction mixture was analyzed by Sepax Bio C-18 column (4.6 × 260 mm, 5 μm particle size) with a gradient of 0.5%–20% of 50 mM TEAA (Triethylammonium acetate, pH 7.5) in ACN in 20 min at 25 ℃ on an Agilent 1200 series HPLC system (Agilent Technologies). Collected HPLC fractions were lyophilized with Labconco FreeZone 2.5 lyophilizer and re-dissolved in autoclaved water. Purified thAMP is subject to quantum yield determination.

PDE-1C-mediated hydrolysis of c^{tz}AMP. c^{tz}AMP (0.1 mM) was incubated with 2 μg/mL PDE1C (Human recombinant full length; BPS Bioscience, Catalog 60013) and 1.08 μg/mL calmodulin (Sigma, Catalog P1431) in a buffer containing 25 mM Tris, pH 7.0, 10 mM $MgCl_2$ and 50 μM $CaCl_2$ at 30 ℃. After 30 min incubation, half of the reaction mixture was directly filtered through a 0.22 μm filter. The rest was quenched with EDTA (20 μL 10 mM) and c^{tz}AMP (0.03 mM) was added before filtration. Both supernatants were subsequently analyzed by Sepax Bio C-18 column (4.6 × 260 mm, 5 μm particle size) with a gradient of 0.5%–20% of 50 mM TEAA (Triethylammonium acetate, pH 7.5) in ACN in 20 min at 25 ℃ on an Agilent 1200 series HPLC system (Agilent Technologies). Collected HPLC fractions were lyophilized with Labconco

FreeZone 2.5 lyophilizer and re-dissolved in autoclaved water. Purified ^{tz}AMP is subject to quantum yield determination.

cAMP-Glo assay luminescence determination. Stock solutions of cAMP (4 μM) and its emissive analogs (4 μM) were prepared and diluted into a separate 96-well, white-bottom plate. The assay was performed using a cAMP-Glo Assay Kit (Promega, V1501), following the manufacturer's protocols. Luminescence (plate without a lid) was measured by Agilent BioTek Synergy H1 multimode microplate reader.

4. Photophysical characterization

Absorption and Emission Spectra of cAMP emissive analogs and corresponding nucleosides. Absorption spectra were acquired on a Shimadzu UV-S450 spectrophotometer, with a 1 nm slit and 1 nm resolution. Steady-state emission spectra were measured on a Horiba Fluoromax-4. Excitation and emission spectra were collected with excitation and emission slits at 1 nm and integration time at 0.1 s. Steady state emission spectra were recorded using excitation 338 nm for ^{tz}A and c^{tz}AMP, 341 nm for thA and cthAMP. All the spectra were blanked against PBS buffer. All the emission spectra were corrected for the instrumental response. Both instruments were equipped with a thermostat-controlled ethylene glycol-water bath to a specially designed cuvette holder.

For temperature-dependent experiments, temperatures were set from 5 to 55 ℃ at 10 ℃ increments. Upon reaching each target temperature, the solution was equilibrated for 20 min in the cuvette holder prior to recording the absorption and emissive spectra. After the completion of the measurement at 55 ℃, the sample was cooled back to 5 ℃, and both absorption and emission spectra were re-measured.

Fluorescence quantum yield determination. Sample concentrations were adjusted to optical density lower than 0.1 at the excitation wavelength (λ_{ex}). Measurements were carried out in Phosphate-Buffered Saline (PBS, 1 ×, without Mg^{2+} and Ca^{2+}). The fluorescence quantum yields (Φ) were determined using Quinine Bisulfate (QBS, 0.546 in 0.5 M H_2SO_4, λ_{ex} 350 nm) as a standard, using the equation:

$$\Phi = \Phi_{STD} \frac{I}{I_{STD}} \frac{OD_{STD}}{OD} \frac{n^2}{n_{std}^2}$$

Where Φ_{STD} is the fluorescence quantum yield of the standard, I and I_{STD} are the integrated area of the emission band of the sample and the standard respectively, OD and OD_{STD} are the optical density at the excitation wavelength for the sample and the standard respectively and n and n_{STD} are the solvent refractive index of the sample and the standard solutions respectively.

To investigate the solvent isotope effect, quantum yield determinations were additionally performed in non-deuterated methanol (CH_3OH) solvent and deuterated methanol (CD_3OD) solvent, with the corresponding solvent refractive indexes in the calculations.

Fluorescence lifetime determination.[4, 5] Briefly, the absorption and emission spectra were first plotted versus energy in wavenumbers ($\varepsilon(\tilde{\nu})$ vs $\tilde{\nu}$ for absorption and normalized photoluminescence vs $\tilde{\nu}$ for emission). The absorption spectrum was then divided by the wavenumber at each point ($\varepsilon(\tilde{\nu})/\tilde{\nu}$), and the radiative lifetime (τ_R) was determined following the Stricker-Berg equation:

$$(\tau_R)^{-1} = \frac{1}{N_A} 8\pi n^2 c \left(2303 \langle \tilde{\nu}_f^{-3} \rangle^{-1}\right) \int \frac{\varepsilon(\tilde{\nu})}{\tilde{\nu}} d\tilde{\nu}$$

where N_A is Avogadro's number, n is the refractive index of the solvent at the mean emission wavelength, c is the speed of light, $\tilde{\nu}_f$ is the emission frequency in wavenumber, and $\langle \tilde{\nu}_f^{-3} \rangle$ is the average of $\tilde{\nu}_f^{-3}$ over the emission spectra weighted by the emission intensity. The fluorescence lifetime (τ_f) was then calculated from the product of the experimental fluorescence quantum yield (Φ_f) and the radiative lifetime (τ_R) calculated by means of the equation described above.

Temperature-dependent NMR spectra. Temperature-dependent NMR spectra were measured on a Bruker 600 MHz spectrometer, equipped with a thermostat-controlled system. c^{th}AMP (2 mM) and c^{tz}AMP (4 mM) were dissolved in D_2O and spectra were taken between 278 and 328 K at 10 K increments. Samples were allowed to equilibrate for 15 min at the desired temperature before data acquisition. After the last spectrum was taken (328 K), the samples were cooled back down to 278 K and the NMR spectra were taken again to ensure compound integrity.

5. Supporting Figures

5.1 AC-mediated Synthesis of ATPs Mixture

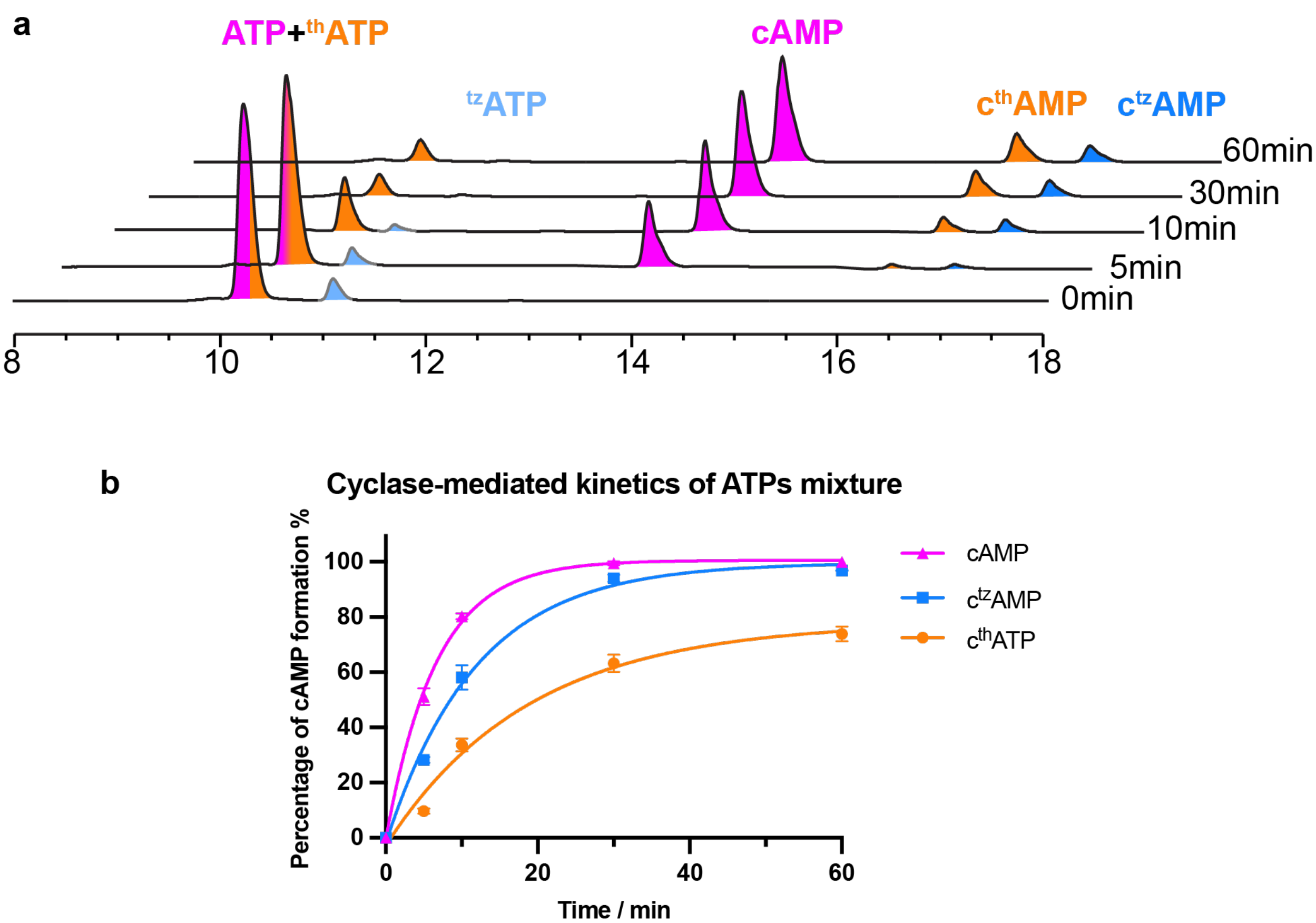

Figure S1. AC-mediated enzymatic reaction of equimolar ATP mixture (ATP: ^{tz}ATP: thATP = 1:1:1) (a) UV-monitored HPLC chromatograms (260 nm) at given time. (b) Normalized time courses of AC-mediated synthesis of cAMP, cthAMP and c^{tz}AMP mixture. Assays were duplicated. Error bars indicate standard deviation (SD).

5.2 Crystal Structures of 3’,5’-cAMP

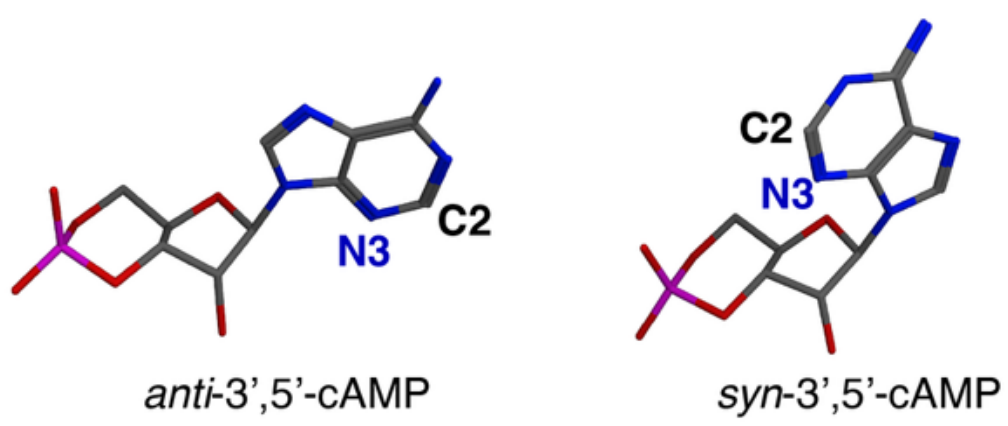

Figure S2. X-ray Crystal structures of *anti*-3’,5’-cAMP and *syn*-3’,5’-cAMP (Cambridge structural database number: 1476478)

5.3 Solvent Isotope Effect of Quantum Yield in thA and ^{tz}A Families

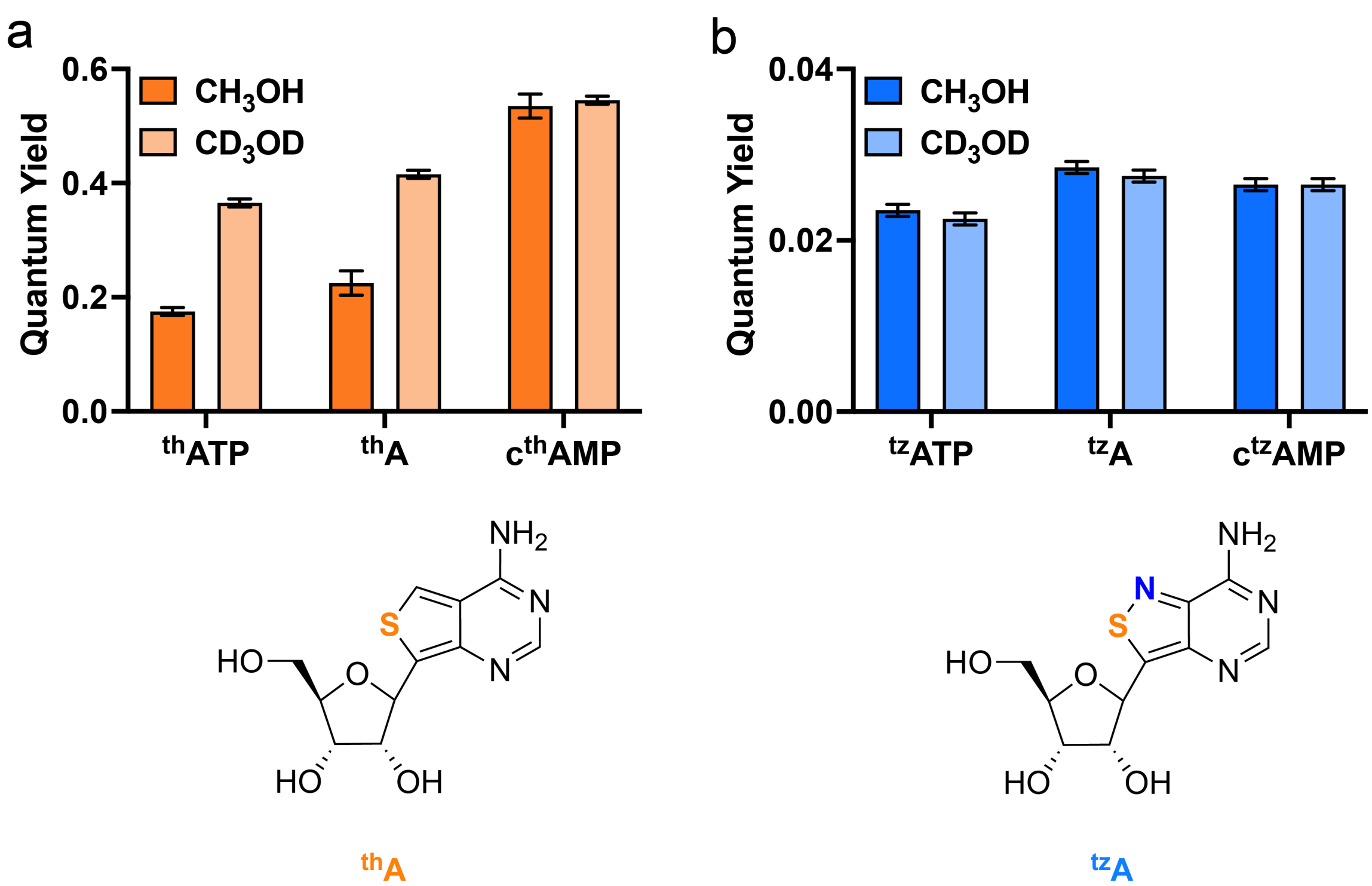

Figure S3. Solvent isotope effect on fluorescence quantum yields (Φ_f). Comparison of Φ_f values recorded in non-deuterated methanol (CH_3OH, dark bars) and deuterated methanol (CD_3OD, light bars) for the (a) thiopheno-adenosine (thA) family and (b) isothiazolo-adenosine (^{tz}A) family.

5.4 Emissive Spectra upon Variable Temperatures of cAMP Analogs and Their Corresponding Nucleosides

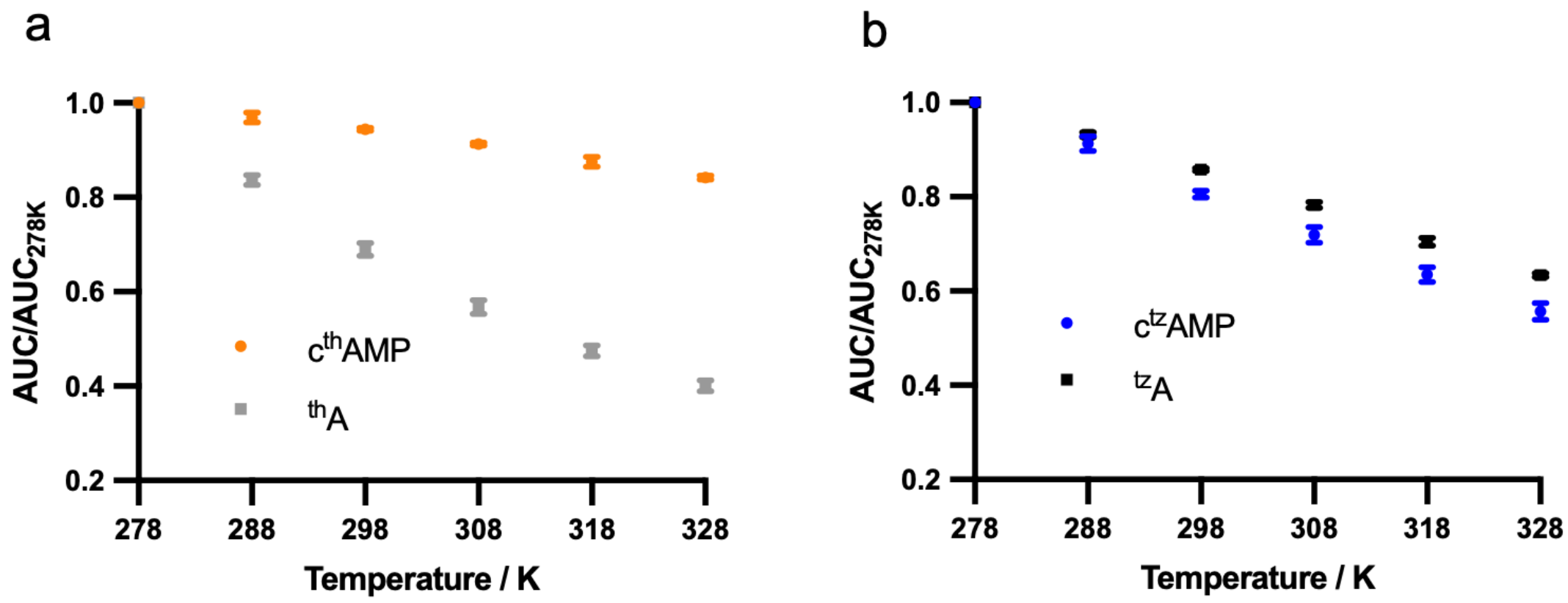

Figure S4. Temperature-dependent relative fluorescence intensity of thA, cthAMP analogs (a), ^{tz}A, c^{tz}AMP analogs (b). Relative fluorescence intensities were obtained by integrating the emission curve (Area Under Curve, AUC) and normalizing to the initial values at 278 K (AUC_{278K}). Assays were done in triplicate. Error bars indicate standard deviation (SD). Note: This figure is a detailed version of Figure 4d, e in the main text, specifically provided here to clearly display the error bars, which are too small to be recognized in the main text version.

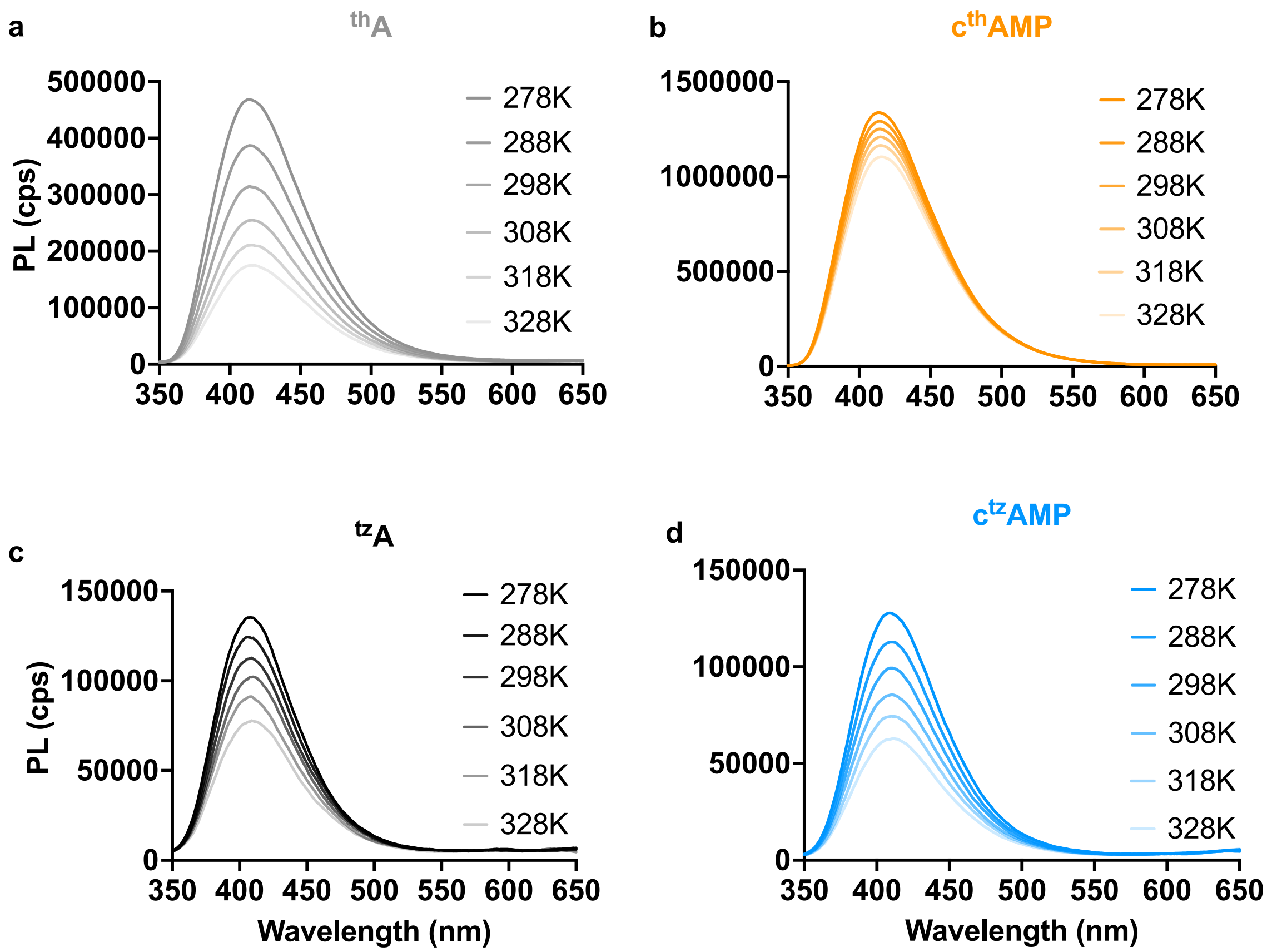

Figure S5. Temperature-dependent emission spectra of thA (a), cthAMP (b), ^{tz}A (c), c^{tz}AMP (d) analogs. Excitation wavelength was 341 nm (a,b), 338 nm (c,d), respectively.

5.5 Crystal Structure of 2',3'-cAMP

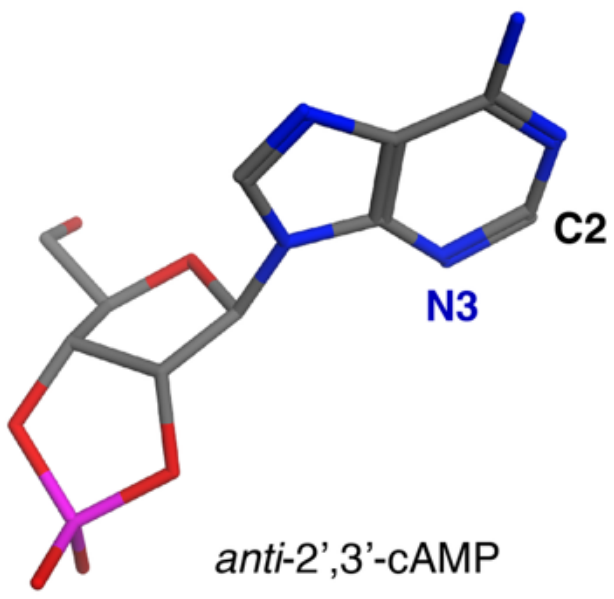

Figure S6. X-ray Crystal structure of *anti*-2',3'-cAMP (Crystallography Open Database ID: 2015424)

5.6 Emission spectra of thAMP and ^{tz}AMP in PBS buffer

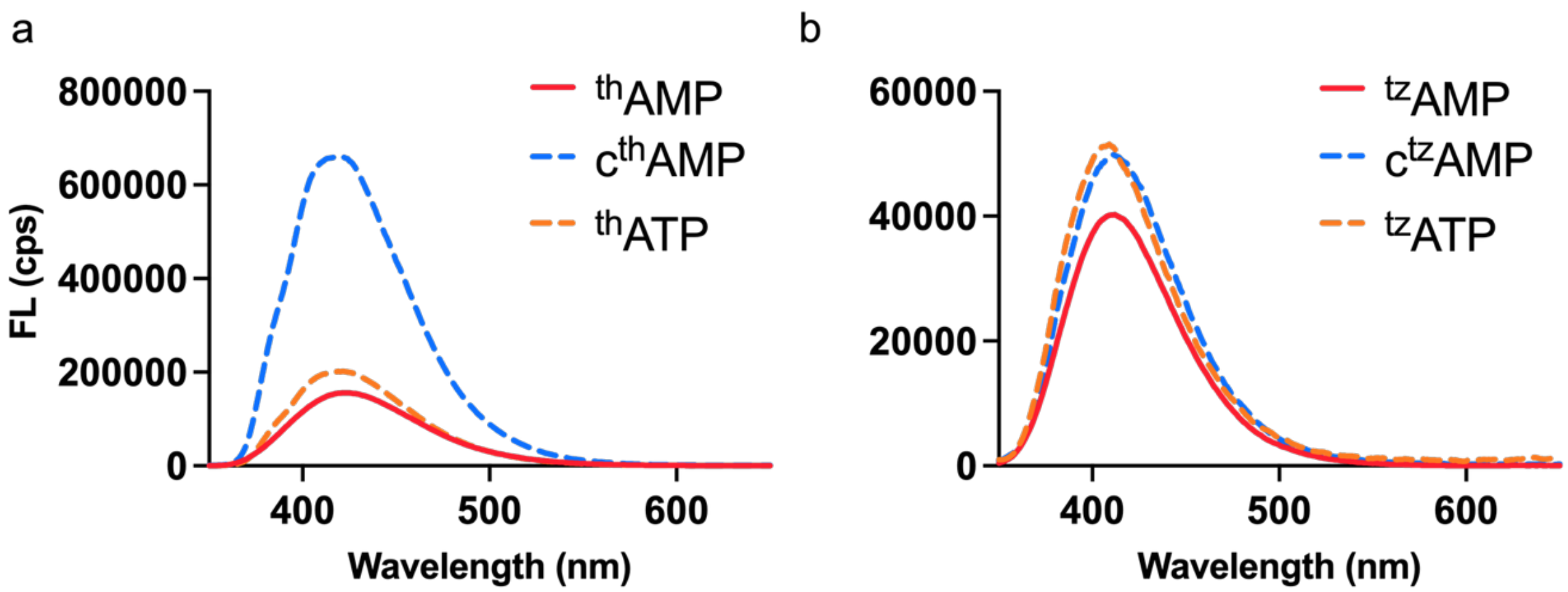

Figure S7. Emission spectra of thAMP (a), and ^{tz}AMP (b) alongside their cyclic and triphosphate counterparts in 1 × PBS buffer, without Mg^{2+} and Ca^{2+}. Excitation wavelength was 341 nm (a), 338 nm (b), respectively.

5.7 X-ray Crystal Structures of PKA-bound cAMP

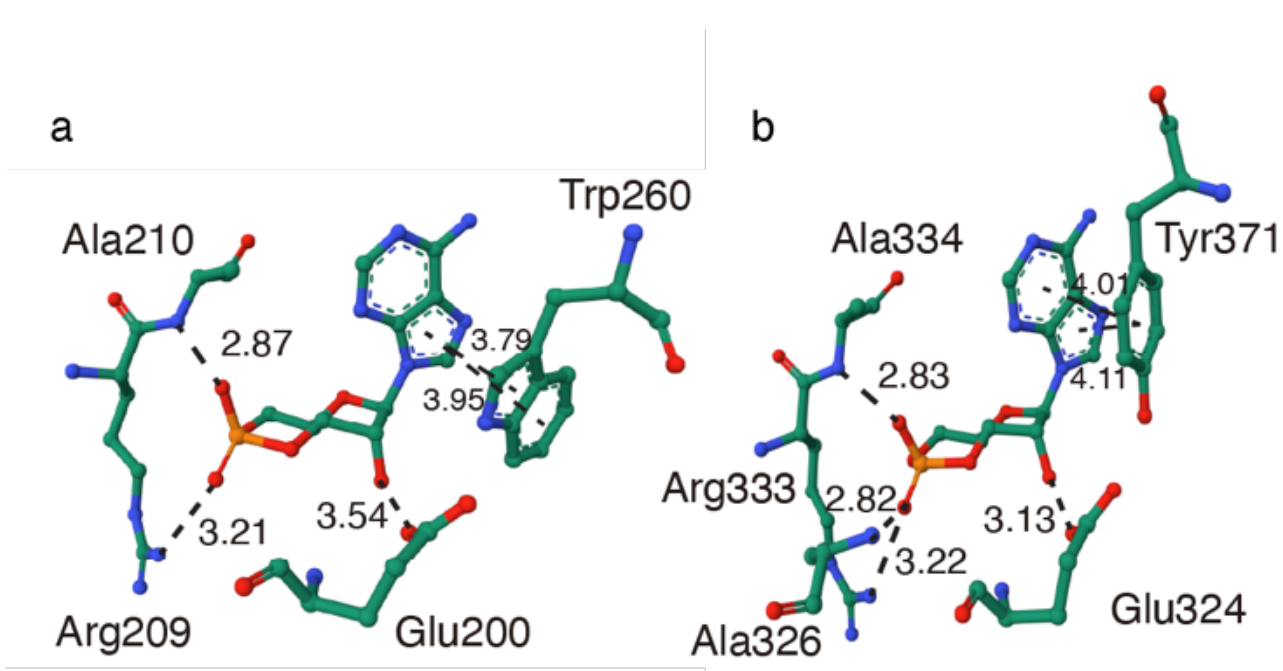

Figure S8. X-ray co-crystal structure of cAMP and PKA (a,b). cAMP binds in two sites of PKA (PDB: 1RGS)

5.8 HPLC Traces of PDE-mediated Hydrolysis

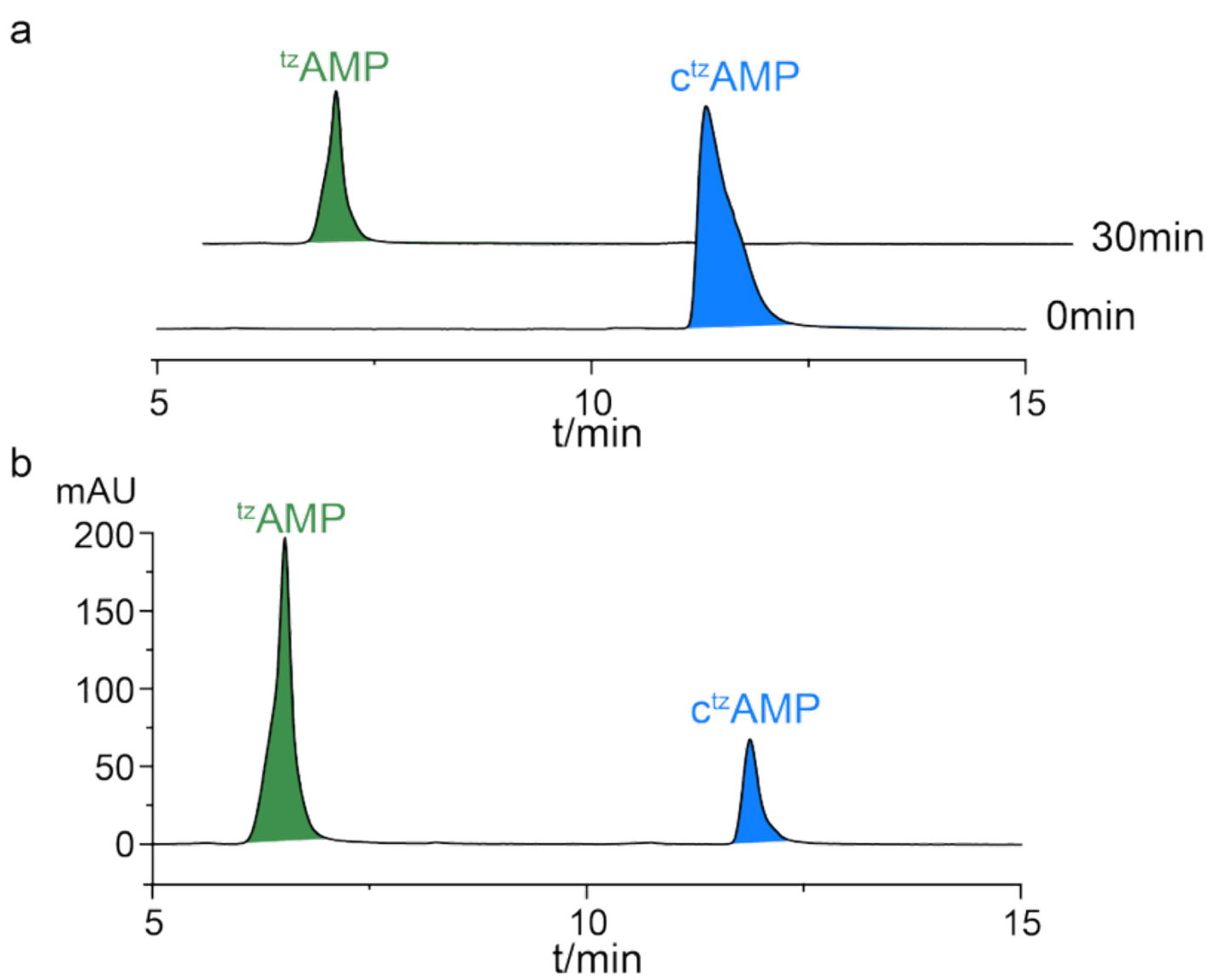

Figure S9. PDE-mediated hydrolysis of c^{tz}AMP. (a) UV-monitored HPLC chromatograms (338 nm) at a given time. (b) UV-monitored HPLC chromatograms (338 nm) of a mixture of ^{tz}AMP and c^{tz}AMP.

5.9 Emission Spectra upon Variable Structures of thA and ^{tz}A Families in CH_3OH and CD_3OD

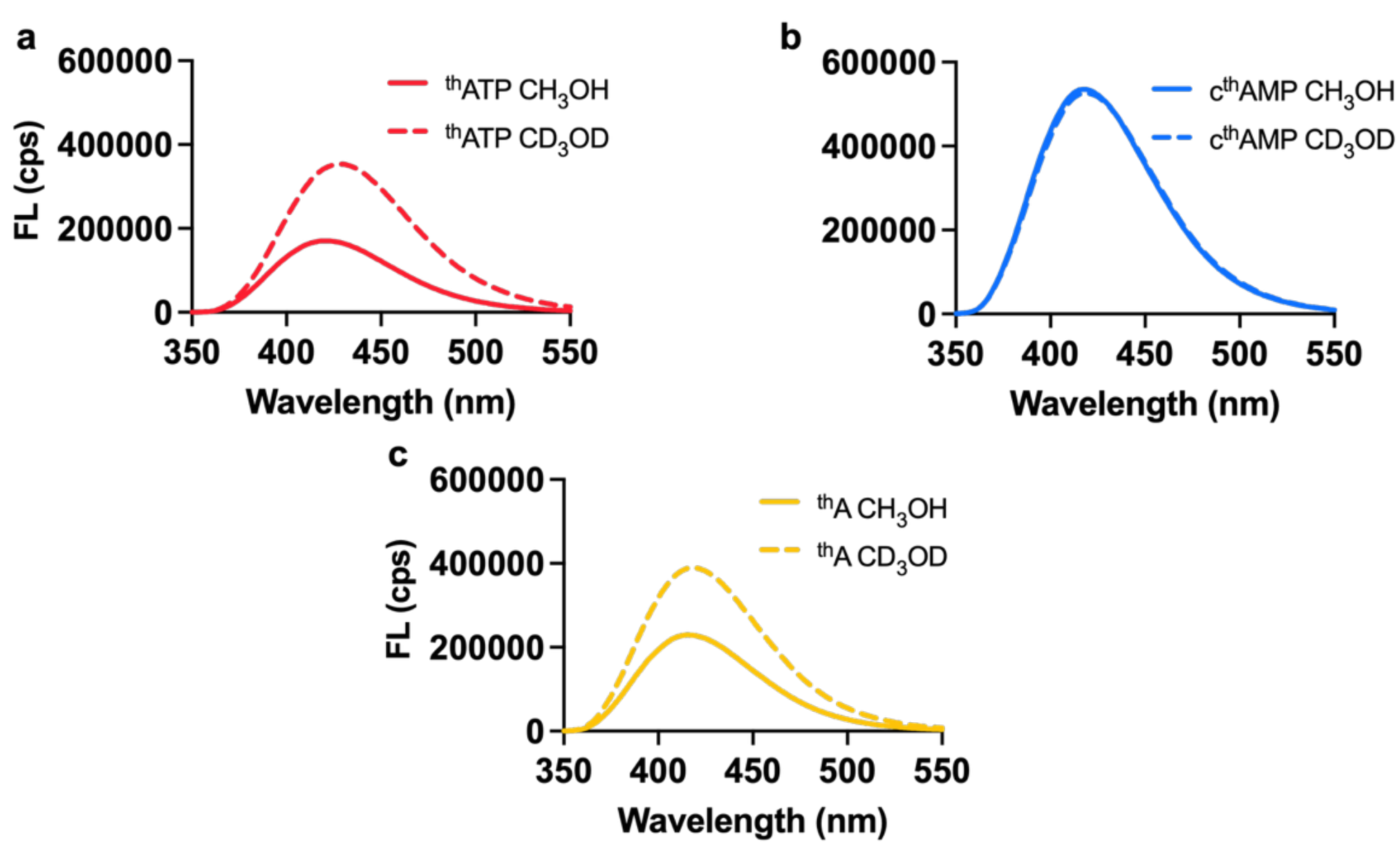

Figure S10. Steady-state emission spectra of the thiopheno-adenosine (thA) derivatives in methanol (CH_3OH, solid line) and deuterated d_4-methanol (CD_3OD, dashed line). (a) thATP, (b) cthAMP, and (c) thA nucleoside.

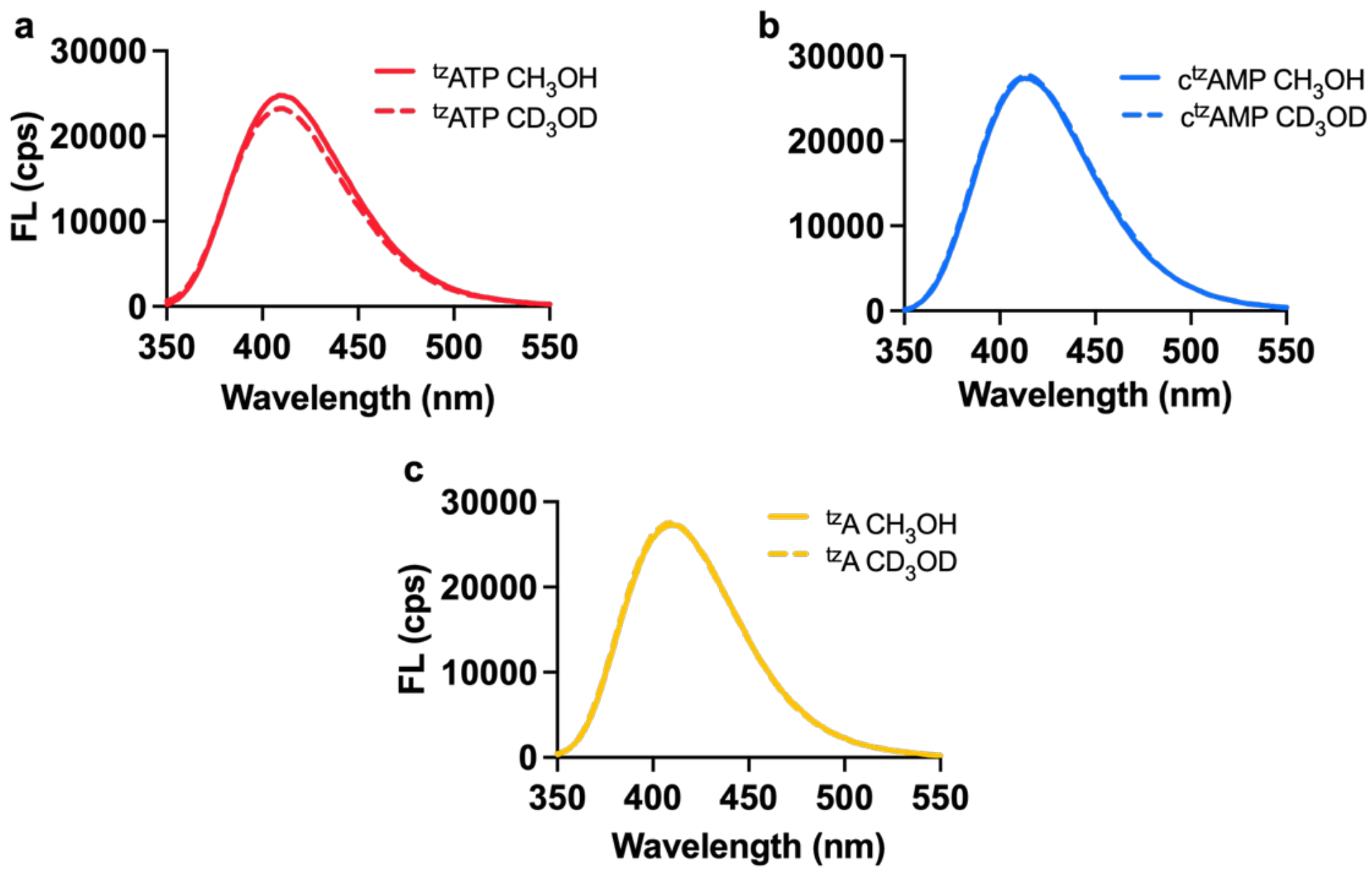

Figure S11. Steady-state emission spectra of the isothiazolo-adenosine (^{tz}A) derivatives in methanol (CH_3OH, solid line) and deuterated d_4-methanol (CD_3OD, dashed line). (a) ^{tz}ATP, (b) c^{tz}AMP, and (c) ^{tz}A nucleoside.

5.10 NMR Spectra of Fluorescent cAMP Analogs

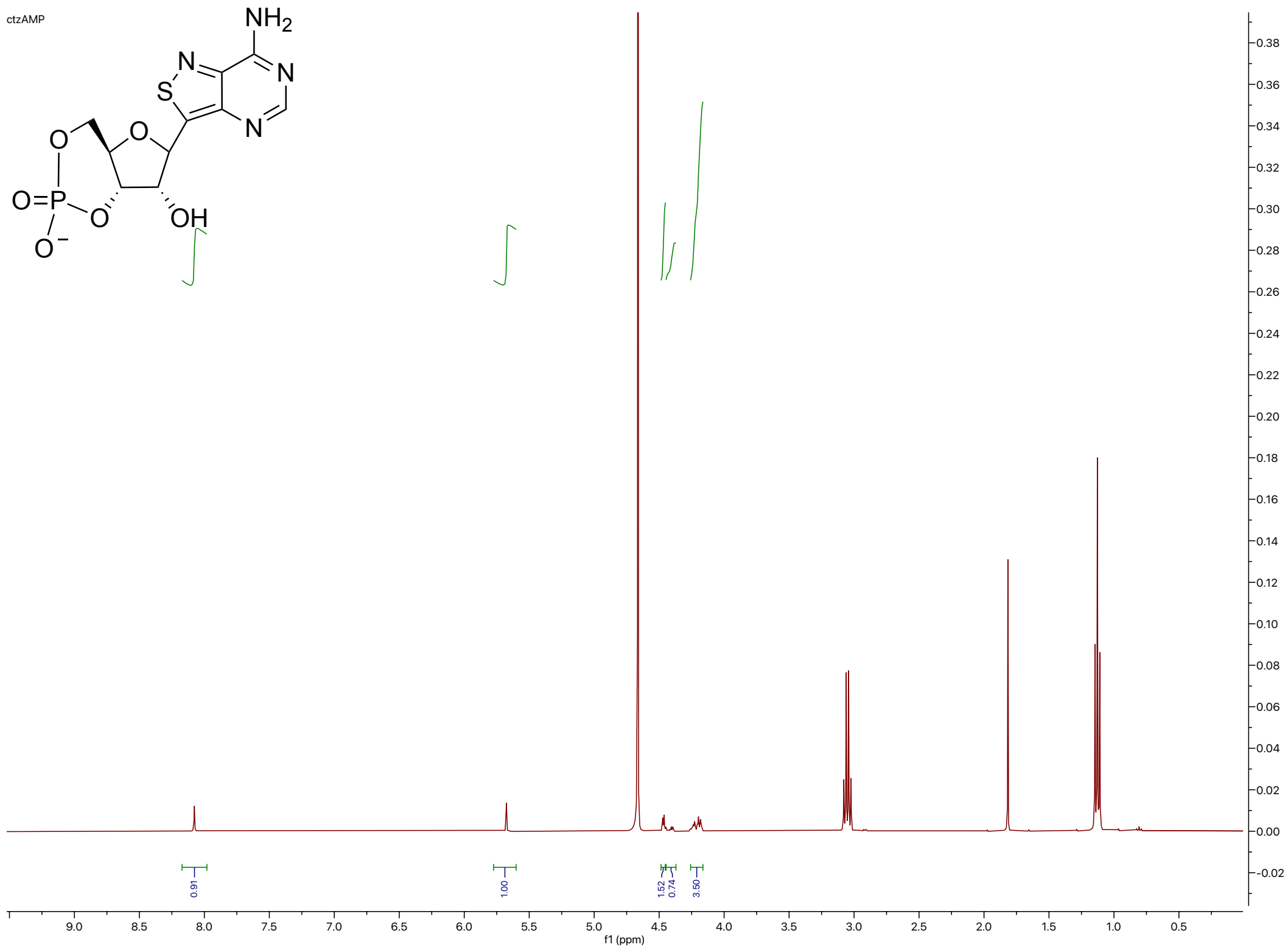

Figure S12. ^{1}H NMR spectrum (400 MHz, D_2O) of c^{tz}AMP. The product was isolated as a triethylammonium (TEA) salt with additional residual triethylammonium acetate (TEAA) salt from the HPLC purification process.

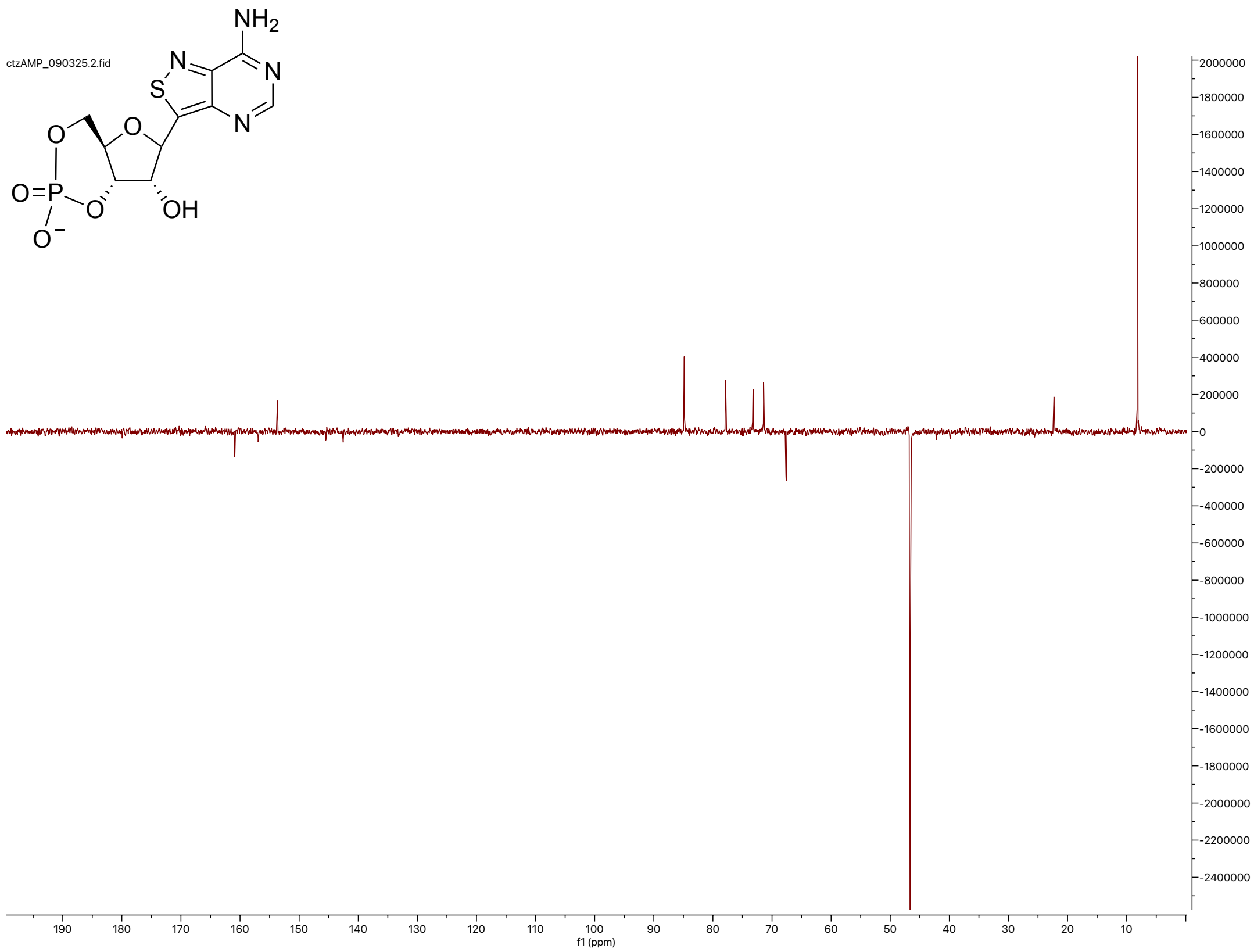

Figure S13. ^{13}C NMR spectrum (151 MHz, D_2O) of c^{tz}AMP. The product was isolated as a triethylammonium (TEA) salt with additional residual triethylammonium acetate (TEAA) buffer from the HPLC purification process.

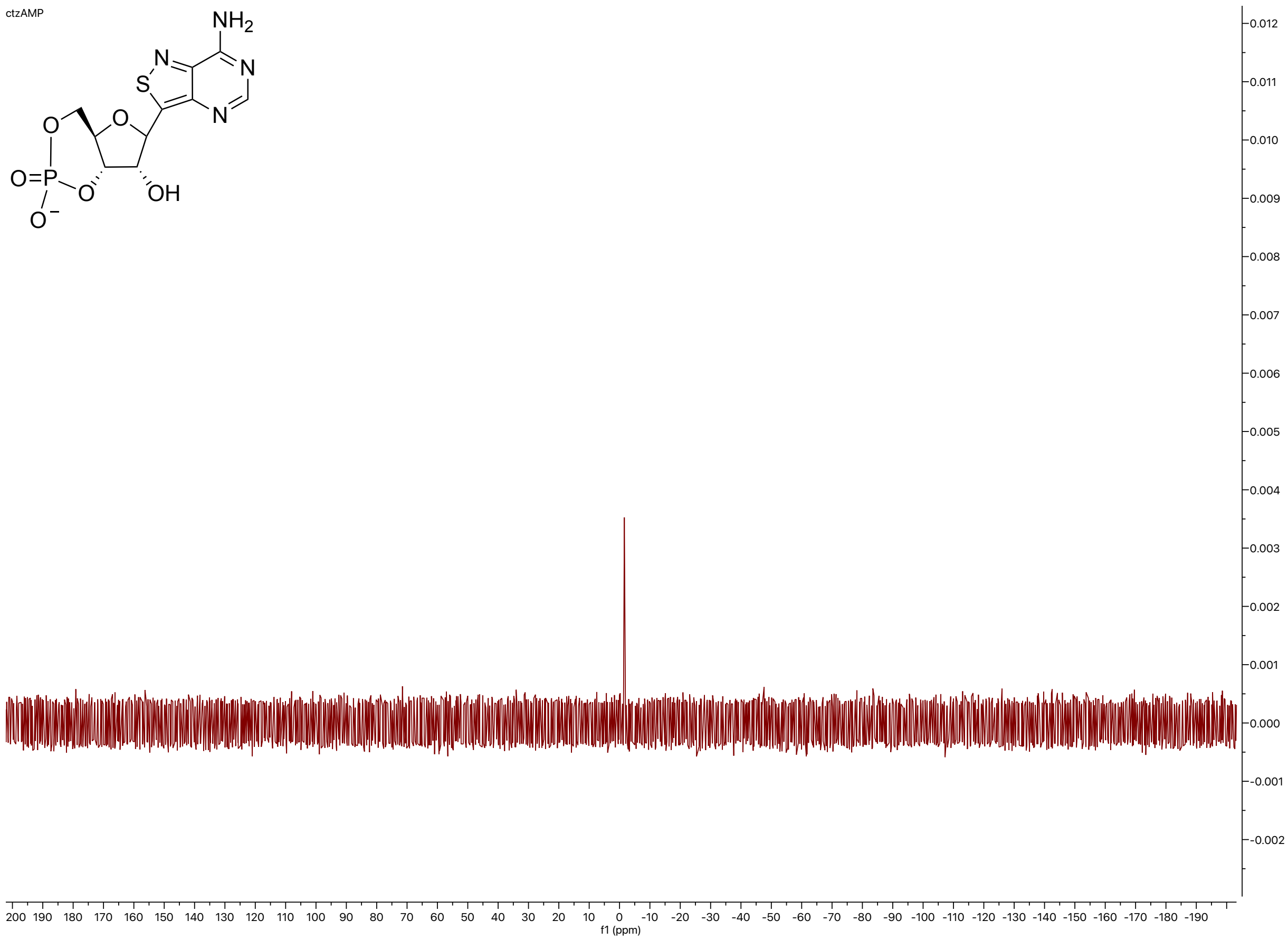

Figure S14. ^{31}P NMR spectrum (162 MHz, D_2O) of c^{tz}AMP. The product was isolated as a triethylammonium (TEA) salt with additional residual triethylammonium acetate (TEAA) buffer from the HPLC purification process.

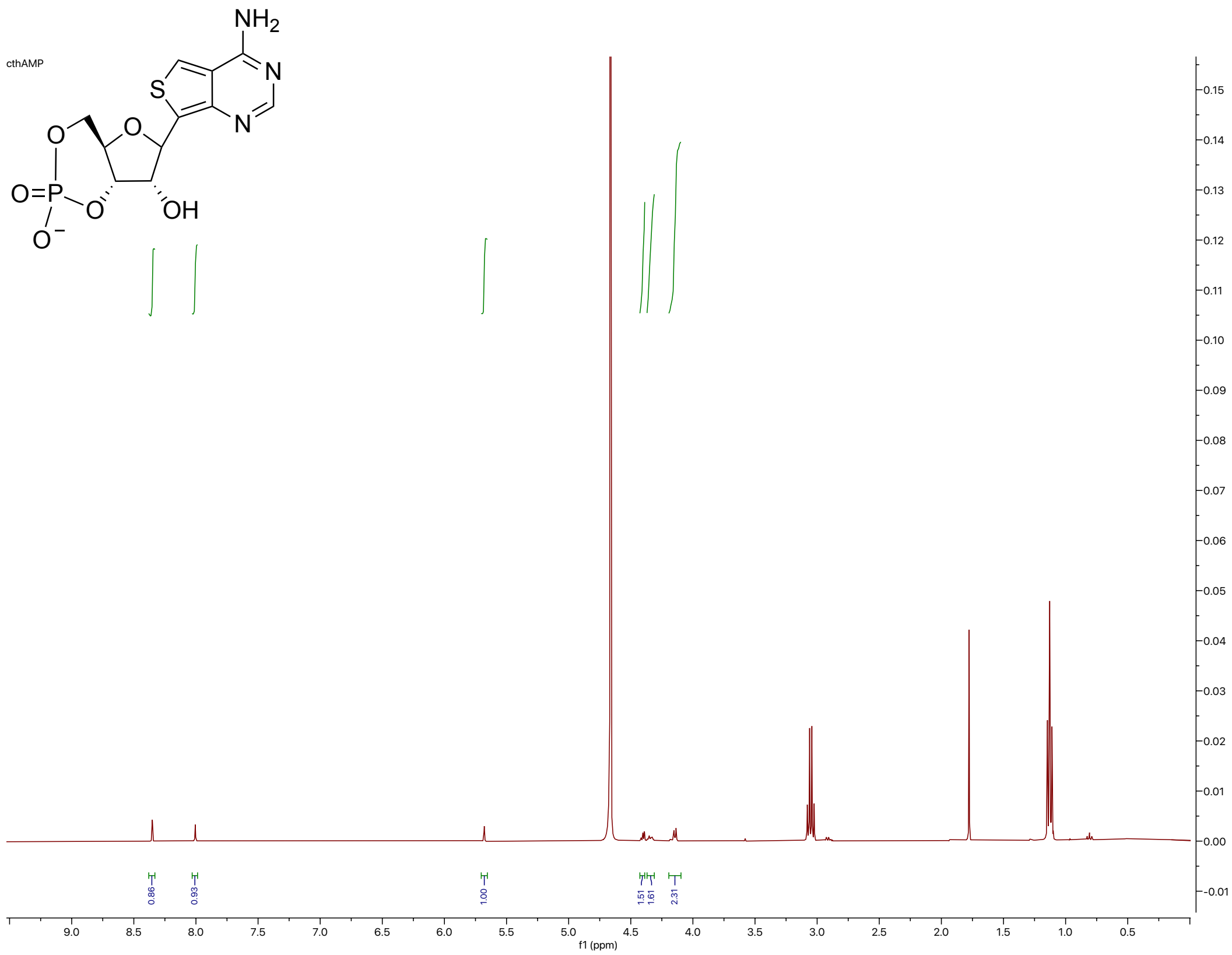

Figure S15. ^{1}H NMR spectrum (400 MHz, D_2O) of cthAMP. The product was isolated as a triethylammonium (TEA) salt with additional residual triethylammonium acetate (TEAA) buffer from the HPLC purification process.

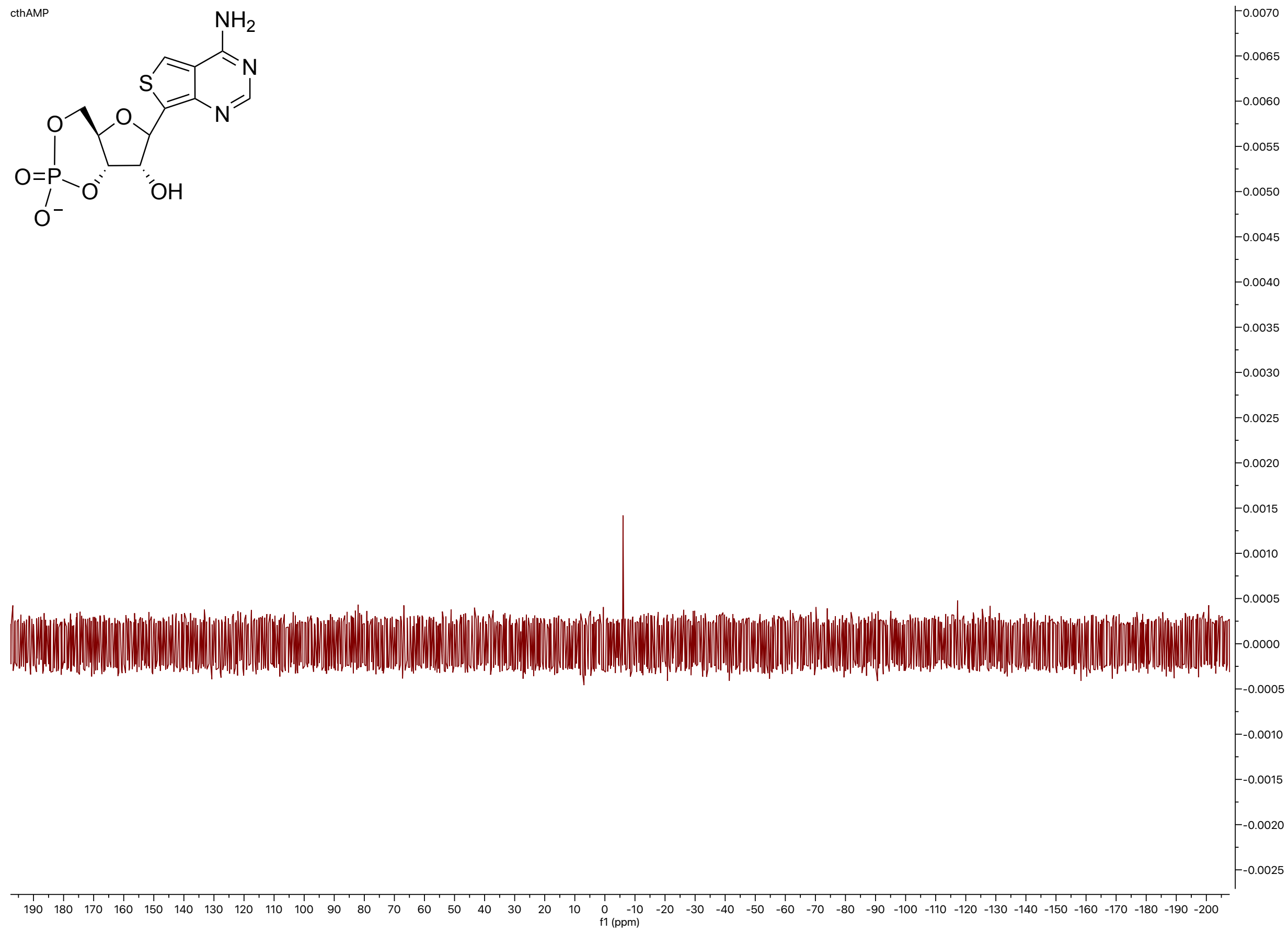

Figure S16. ^{31}P NMR spectrum (162 MHz, D_2O) of cthAMP. The product was isolated as a triethylammonium (TEA) salt with additional residual triethylammonium acetate (TEAA) buffer from the HPLC purification process.

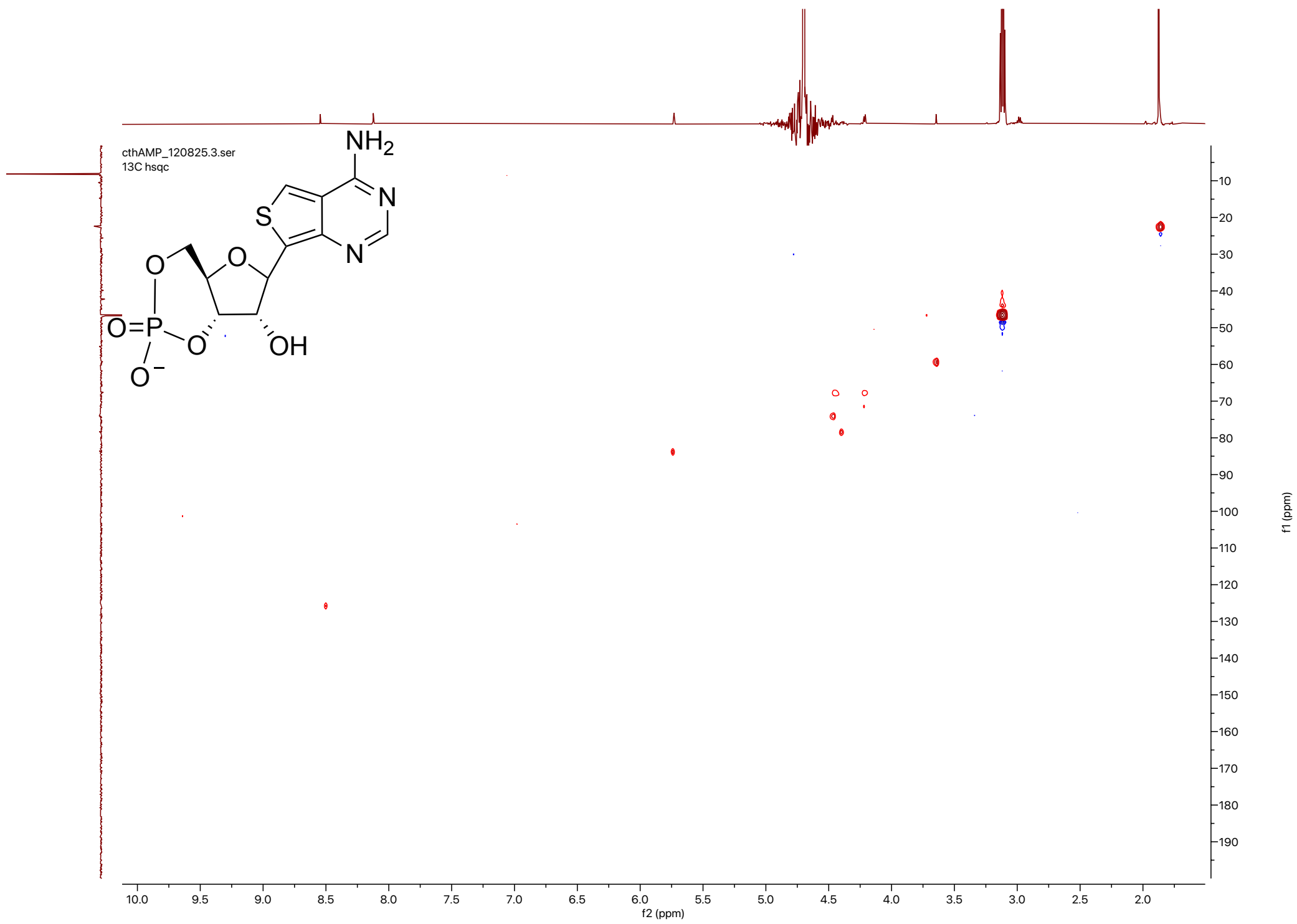

Figure S17. ^{1}H-^{13}C HSQC NMR spectrum (600 MHz, D_2O) of cthAMP. The product was isolated as a triethylammonium (TEA) salt with additional residual triethylammonium acetate (TEAA) buffer from the HPLC purification process.

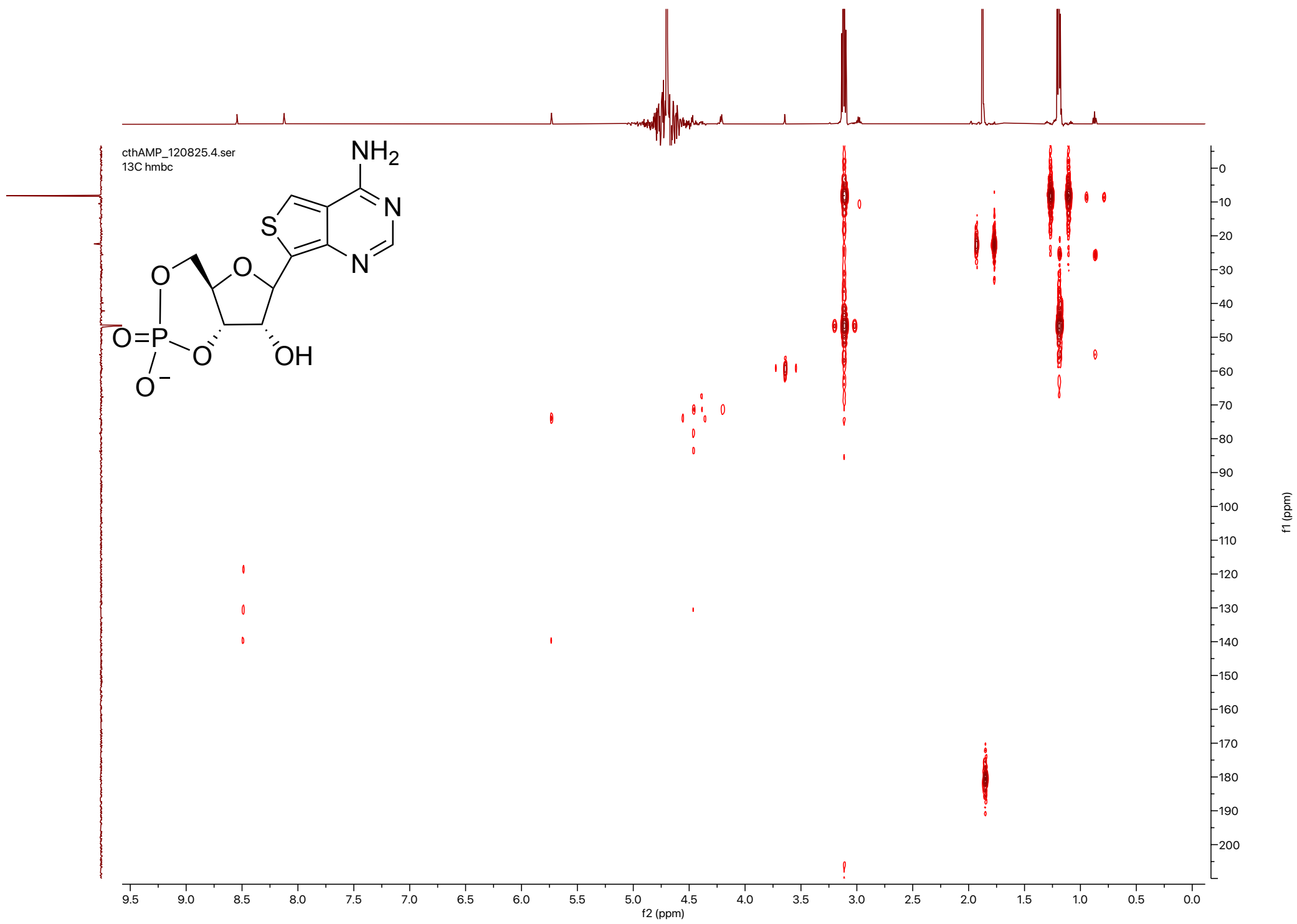

Figure S18. ^{1}H-^{13}C HMBC NMR spectrum (600 MHz, D_2O) of cthAMP. The product was isolated as a triethylammonium (TEA) salt with additional residual triethylammonium acetate (TEAA) buffer from the HPLC purification process.

6. Supporting Tables

Table S1. Calculated photophysical parameters for emissive cAMP analogs and their precursors

	ε [M^{-1} cm^{-1}]	Φ_f	τ_f [a][ns]	k_r [a][s^{-1}]	k_{nr} [a][s^{-1}]
thAMP	7.4×10^3	0.19	2.63	7.21×10^7	3.07×10^8
thATP	7.4×10^3	0.21	2.90	7.25×10^7	2.73×10^8
cthAMP	7.4×10^3	0.67	9.93	6.75×10^7	3.32×10^7
tzAMP	7.8×10^3	0.040	0.52	7.72×10^7	1.85×10^9
tzATP	7.8×10^3	0.053	0.70	7.56×10^7	1.35×10^9
ctzAMP	7.8×10^3	0.051	0.69	7.32×10^7	1.36×10^9

[a] Fluorescence lifetimes were calculated according to the Strickler–Berg equation,[4, 5] allowing us to extract radiative (k_r) and non-radiative decay rate constants (k_{nr}).

Table S2. Solvent isotope effect on fluorescence quantum yields (Φ_f) of thiopheno- and isothiazolo-adenosine derivatives in CH_3OH and CD_3OD

Solvent	Thiopheno-adenosine (thA) family Quantum Yield (Φ_f)			Isothiazolo-adenosine (tzA) family Quantum Yield (Φ_f)		
	**thATP**	**thA**	**cthAMP**	**tzATP**	**tzA**	**ctzAMP**
**CH_3OH**	0.18±0.01	0.23±0.02	0.53±0.02	0.024±0.001	0.029±0.001	0.027±0.001
**CD_3OD**	0.37±0.01	0.42±0.01	0.54±0.01	0.023±0.001	0.028±0.001	0.027±0.001

[a] Quantum yield values (Φ_f) are presented as mean ± standard deviation (SD). Measurements were duplicated.

7. Computational Analysis of cAMP Analogs

7.1 Geometry Optimization

Geometry optimizations of the ground state (S_0) and the first excited singlet state (S_1) were performed using B3LYP combined with 6-31+G(d,p) as the basis set. These geometries were optimized in water, and solvent effects were accounted for using the IEFPCM solvation model. A frequency calculation was done for each geometry to ensure the minima were found. Ground-state and excited-state populations were calculated using the relationship between Gibbs free energy and the equilibrium constant K. For each species, the Gibbs free energy was obtained by adding the thermal correction to Gibbs free energy obtained by the frequency calculation to the electronic energy. In each case, these energies were computed by applying a correction using Truhlar's quasiharmonic oscillator approximation,[6] which is included in the GoodVibes program.[7] The frequency cut-off was set to 200 cm^{-1}.

Anti - thAMP – B3LYP/6-31+G(d,p) – IEFPCM Water

Ground state

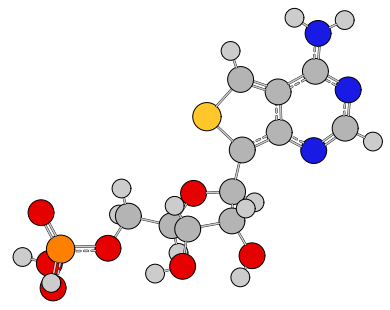

Cartesian coordinates

Atom	X	Y	Z
C	1.926211	-0.014896	0.245012
C	3.295833	0.212414	0.274540
C	4.057071	-0.940072	-0.147426
S	1.596136	-1.631820	-0.286007
C	5.495778	-0.788490	-0.143480
C	5.210750	1.377388	0.609753
H	5.720119	2.291287	0.909530
N	6.043873	0.365545	0.231650
N	3.898011	1.391985	0.657926
N	6.310883	-1.795404	-0.513295
H	7.309913	-1.648388	-0.502877
H	5.961181	-2.691030	-0.814471
C	0.841506	0.961105	0.580078
H	1.242849	1.685047	1.297775
C	0.252099	1.765885	-0.609562
H	0.877969	1.687281	-1.504787
C	-1.134775	1.130985	-0.806087
H	-1.042988	0.229846	-1.427425
C	-1.502699	0.732167	0.632139
H	-1.855684	1.618612	1.178353
O	0.129977	3.129233	-0.210743
H	-0.618268	3.504715	-0.703482
O	-2.004631	2.073743	-1.414142
H	-2.912438	1.740960	-1.347755
O	-0.274848	0.248848	1.179047
C	-2.530442	-0.383450	0.743098
H	-2.759637	-0.608809	1.787998
H	-2.180864	-1.286496	0.235315
C	3.258691	-2.015693	-0.484750
H	3.538571	-3.006344	-0.815613
O	-3.724580	0.116727	0.077557
P	-5.058158	-0.777593	0.005654
O	-4.887698	-2.177547	-0.466202
O	-6.024412	0.159087	-0.868329
H	-5.976791	-0.016807	-1.821767
O	-5.649968	-0.598376	1.488000
H	-6.269945	-1.299504	1.745157

Excited state

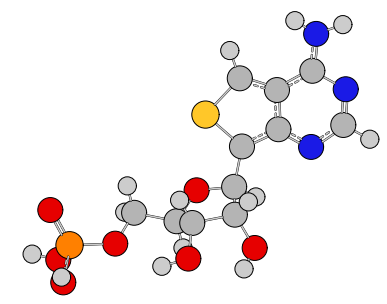

Cartesian coordinates

Atom	X	Y	Z
C	1.901113	-0.060113	0.327425
C	3.295443	0.192705	0.294972
C	4.056694	-0.959197	-0.133727
S	1.513164	-1.717370	-0.129444
C	5.460767	-0.784684	-0.217335
C	5.233041	1.395548	0.505575
H	5.697907	2.344177	0.763038
N	6.037226	0.422990	0.136718
N	3.863648	1.366901	0.620035
N	6.326725	-1.762208	-0.607475
H	7.279386	-1.481305	-0.800078
H	5.990673	-2.573486	-1.107479
C	0.848955	0.935694	0.646137
H	1.264109	1.663241	1.356316
C	0.291833	1.756373	-0.560475
H	0.935657	1.673189	-1.441956
C	-1.094393	1.127220	-0.782147
H	-0.990720	0.221553	-1.395124
C	-1.501549	0.744147	0.649318
H	-1.863897	1.636879	1.178033
O	0.173779	3.115377	-0.157019
H	-0.562910	3.499881	-0.660623
O	-1.941594	2.072479	-1.416359
H	-2.854689	1.751326	-1.364019
O	-0.289763	0.263214	1.238112
C	-2.536345	-0.366400	0.744766
H	-2.788282	-0.583738	1.786079
H	-2.181021	-1.274409	0.250009
C	3.235860	-2.051313	-0.387009
H	3.521937	-3.051293	-0.686470
O	-3.712687	0.136791	0.051500
P	-5.048745	-0.752224	-0.046081
O	-4.876010	-2.150613	-0.521459
O	-5.996310	0.192161	-0.931857
H	-5.942263	0.012060	-1.884138
O	-5.661904	-0.576470	1.427905
H	-6.291128	-1.273981	1.672212

Syn - thAMP – B3LYP/6-31+G(d,p) – IEFPCM Water

Ground state

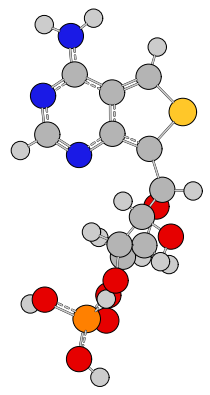

Excited state

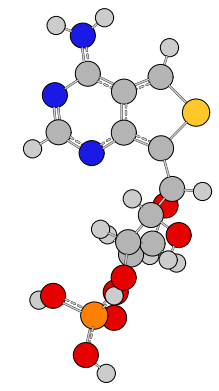

	Cartesian coordinates (Ground state)				Cartesian coordinates (Excited state)		
**Atom**	**X**	**Y**	**Z**	**Atom**	**X**	**Y**	**Z**
C	-2.207481	1.008470	-0.398245	C	-2.218857	1.034046	-0.405400
C	-2.587393	-0.221285	0.131090	C	-2.583514	-0.238792	0.110679
C	-3.920678	-0.607125	-0.265850	C	-3.922971	-0.631988	-0.268811
S	-3.487891	1.651369	-1.375692	S	-3.514313	1.736112	-1.373056
C	-4.399820	-1.871221	0.248093	C	-4.372042	-1.882857	0.228098
C	-2.379870	-2.130571	1.335965	C	-2.344919	-2.198993	1.278439
H	-1.792466	-2.777078	1.985342	H	-1.703046	-2.819881	1.898495
N	-3.620357	-2.608924	1.036730	N	-3.539232	-2.671506	1.001727
N	-1.810756	-1.010577	0.950301	N	-1.789685	-1.007940	0.872529
N	-5.628100	-2.335084	-0.053148	N	-5.602483	-2.398529	-0.025872
H	-5.929057	-3.217066	0.335959	H	-5.879841	-3.229377	0.479468
H	-6.274752	-1.820414	-0.629426	H	-6.340206	-1.825969	-0.410034
C	-0.912183	1.746529	-0.264762	C	-0.926088	1.752433	-0.249214
H	-1.038343	2.772716	-0.628439	H	-1.031236	2.784909	-0.599859
C	-0.314227	1.850515	1.164174	C	-0.333026	1.826525	1.190101
H	-1.002541	1.475198	1.926461	H	-1.023333	1.428828	1.938969
C	0.966782	1.002343	1.080021	C	0.953634	0.984371	1.094917
H	0.714352	-0.041281	1.297879	H	0.707661	-0.061678	1.306281
C	1.354422	1.142072	-0.400256	C	1.341749	1.137393	-0.383628
H	1.860363	2.106807	-0.558147	H	1.844099	2.105401	-0.532844
O	-0.004406	3.222434	1.414909	O	-0.034440	3.192197	1.473408
H	0.768829	3.234551	2.002984	H	0.760525	3.197577	2.032647
O	1.926736	1.503183	2.002881	O	1.910691	1.485531	2.020287
H	2.779445	1.084334	1.812334	H	2.767146	1.076152	1.825057
O	0.105146	1.096164	-1.087668	O	0.093714	1.096366	-1.075348
C	2.222084	0.014864	-0.938543	C	2.213141	0.017210	-0.930388
H	2.478522	0.172095	-1.988906	H	2.471744	0.185440	-1.978445
H	1.719875	-0.949366	-0.816600	H	1.713326	-0.949439	-0.818685
C	-4.529712	0.322449	-1.088827	C	-4.534242	0.313679	-1.079886
H	-5.515910	0.310304	-1.531926	H	-5.508188	0.274164	-1.549765
O	3.435393	0.032263	-0.132269	O	3.424034	0.030303	-0.120479
P	4.663995	-0.946281	-0.467467	P	4.665678	-0.925006	-0.475751
O	5.750094	-0.484071	0.620333	O	5.747025	-0.468719	0.619127
H	6.319990	0.240369	0.315899	H	6.305723	0.269747	0.327839
O	5.113541	-0.972675	-1.884901	O	5.112688	-0.917782	-1.894170
O	4.149555	-2.342639	0.137614	O	4.171032	-2.339305	0.103299
H	4.612507	-3.116289	-0.221884	H	4.644130	-3.099653	-0.271068

Anti - [tz]AMP – B3LYP/6-31+G(d,p) – IEFPCM Water

Ground state

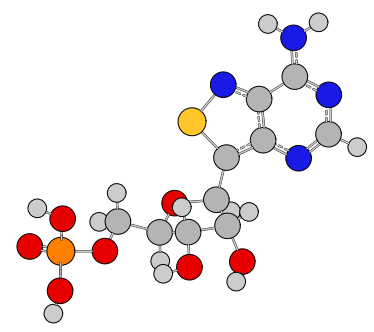

Excited state

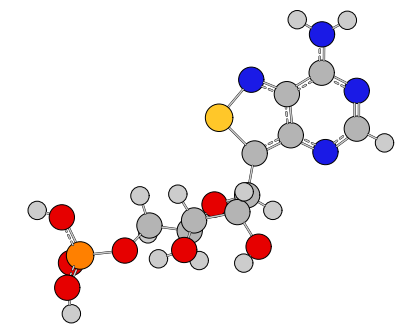

Cartesian coordinates (Ground state)				Cartesian coordinates (Excited state)			
**Atom**	**X**	**Y**	**Z**	**Atom**	**X**	**Y**	**Z**
C	1.892923	0.016399	0.536014	C	1.909769	0.019688	0.219658
C	3.222937	0.259866	0.196783	C	3.297309	0.225399	0.322606
C	3.882303	-0.976555	-0.089771	C	4.032091	-0.927173	-0.138718
S	1.624115	-1.678920	0.475951	S	1.640721	-1.584903	-0.444950
C	5.278120	-0.894333	-0.468124	C	5.451192	-0.826405	-0.095001
C	5.124924	1.401019	-0.220847	C	5.291800	1.305732	0.761176
H	5.669608	2.340527	-0.283375	H	5.778833	2.217322	1.093776
N	5.866746	0.297662	-0.524242	N	6.066516	0.306710	0.386049
N	3.858405	1.477283	0.131129	N	3.928516	1.297496	0.838404
N	5.967973	-2.008347	-0.757981	N	6.248782	-1.799612	-0.565801
H	6.939354	-1.946029	-1.026778	H	7.250130	-1.658323	-0.595127
H	5.522066	-2.912964	-0.721876	H	5.853428	-2.616729	-1.011945
C	0.832937	1.002085	0.922836	C	0.838896	1.004925	0.506431
H	1.218524	1.655682	1.714603	H	1.250282	1.776187	1.170604
C	0.321024	1.901315	-0.229487	C	0.217732	1.743369	-0.718612
H	1.010532	1.910522	-1.079690	H	0.823244	1.612822	-1.621714
C	-1.033479	1.260570	-0.577603	C	-1.171046	1.095276	-0.846365
H	-0.875208	0.411155	-1.255274	H	-1.088266	0.160199	-1.417352
C	-1.506579	0.751773	0.793670	C	-1.508246	0.778265	0.619360
H	-1.935285	1.586100	1.365843	H	-1.851008	1.694135	1.121690
O	0.141693	3.218433	0.280857	O	0.105520	3.124657	-0.392435
H	-0.576757	3.627322	-0.229420	H	-0.649240	3.476594	-0.892752
O	-1.871543	2.235315	-1.178046	O	-2.055983	1.999831	-1.488242
H	-2.773821	1.883879	-1.218076	H	-2.960870	1.667275	-1.386459
O	-0.312874	0.270615	1.420822	O	-0.267776	0.329422	1.169032
C	-2.501194	-0.397488	0.740208	C	-2.532018	-0.329626	0.813334
H	-2.789951	-0.722251	1.742537	H	-2.745341	-0.497185	1.871588
H	-2.089478	-1.241712	0.179072	H	-2.187983	-1.259460	0.350510
O	-3.663919	0.130050	0.042800	O	-3.736938	0.131388	0.139429
N	3.170676	-2.097463	0.013348	N	3.327281	-1.998420	-0.512922
P	-4.985756	-0.765055	-0.145935	P	-5.097612	-0.721874	0.209418
O	-5.994110	0.286625	-0.818168	O	-6.122107	0.244621	-0.559459
H	-6.494020	0.812926	-0.173463	H	-6.540810	0.905280	0.015689
O	-5.471974	-1.474581	1.067032	O	-5.506500	-1.189780	1.560399
O	-4.581967	-1.691564	-1.393984	O	-4.828999	-1.867015	-0.884037
H	-5.117595	-2.497589	-1.468631	H	-5.380022	-2.656473	-0.761413

Syn - ^{tz}AMP – B3LYP/6-31+G(d,p) – IEFPCM Water

Ground state

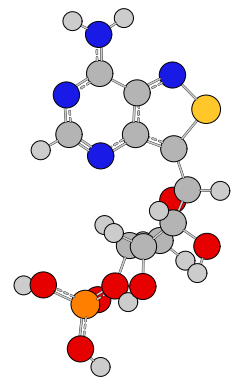

Cartesian coordinates

Atom	X	Y	Z
C	-2.195492	0.992892	-0.413328
C	-2.524781	-0.250053	0.133043
C	-3.861990	-0.602114	-0.234613
S	-3.554701	1.552918	-1.302543
C	-4.364527	-1.866617	0.261439
C	-2.321325	-2.174637	1.297599
H	-1.731841	-2.842013	1.922556
N	-3.576470	-2.626080	1.018288
N	-1.737570	-1.056841	0.918267
N	-5.609260	-2.266649	-0.039273
H	-5.955620	-3.147073	0.313815
H	-6.212250	-1.690333	-0.607393
C	-0.916550	1.773143	-0.332632
H	-1.071921	2.777226	-0.742767
C	-0.318697	1.949415	1.088611
H	-1.010394	1.623186	1.871303
C	0.952815	1.082214	1.045624
H	0.688792	0.052640	1.311466
C	1.344187	1.150465	-0.438780
H	1.864863	2.098610	-0.639193
O	0.005011	3.327390	1.261815
H	0.779881	3.365453	1.846854
O	1.915012	1.617322	1.944742
H	2.761270	1.172726	1.786286
O	0.094100	1.097585	-1.129220
C	2.191900	-0.014263	-0.926646
H	2.450786	0.093316	-1.982672
H	1.673381	-0.963575	-0.763404
O	3.402951	0.018709	-0.119191
P	4.632444	-0.966484	-0.432915
O	5.712870	-0.488075	0.653197
H	6.287377	0.228816	0.339684
O	5.088726	-1.014813	-1.847514
N	-4.544626	0.253317	-0.995101
O	4.112701	-2.352500	0.190708
H	4.575509	-3.132260	-0.155593

Excited state

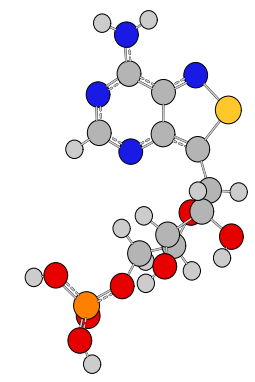

Cartesian coordinates

Atom	X	Y	Z
C	-2.233036	1.013559	-0.442638
C	-2.271790	-0.329200	-0.092747
C	-3.630769	-0.839824	-0.085477
S	-3.900697	1.545524	-0.713231
C	-3.787904	-2.203084	0.308537
C	-1.491100	-2.452196	0.587997
H	-0.640851	-3.059752	0.871464
N	-2.709934	-2.977566	0.589122
N	-1.277473	-1.207438	0.145082
N	-5.026085	-2.749499	0.441496
H	-5.112442	-3.619378	0.950534
H	-5.819462	-2.122716	0.467371
C	-1.062552	1.934530	-0.517225
H	-1.360323	2.857662	-1.029775
C	-0.422536	2.335631	0.835023
H	-1.115219	2.194107	1.671288
C	0.800161	1.411405	0.917648
H	0.487869	0.416770	1.265773
C	1.229775	1.326770	-0.557135
H	1.820053	2.217433	-0.815371
O	-0.016554	3.700171	0.751257
H	0.734892	3.814188	1.355874
O	1.762934	1.975151	1.793827
H	2.588359	1.475012	1.705607
O	0.002520	1.306340	-1.289305
C	2.007876	0.070440	-0.917145
H	2.286711	0.061252	-1.973529
H	1.423217	-0.821975	-0.675126
O	3.208860	0.103073	-0.094145
N	-4.592183	0.002295	-0.454100
P	4.361948	-0.999934	-0.278823
O	5.469174	-0.486745	0.763721
H	6.102792	0.140852	0.380181
O	4.822738	-1.239896	-1.672347
O	3.732782	-2.264904	0.485867
H	4.137691	-3.111079	0.236530

Anti - c-thAMP – B3LYP/6-31+G(d,p) – IEFPCM Water

Ground state 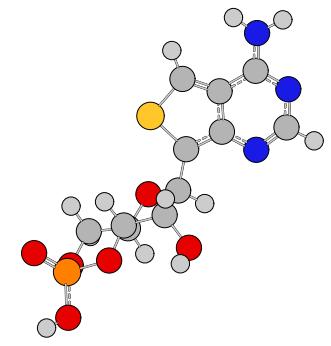

Excited state 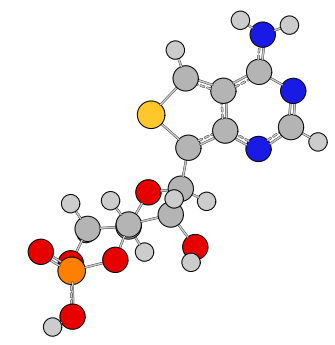

	Ground state: Cartesian coordinates				Excited state: Cartesian coordinates		
**Atom**	**X**	**Y**	**Z**	**Atom**	**X**	**Y**	**Z**
C	1.542457	-0.107256	0.164848	C	1.524954	-0.163976	0.340306
C	2.837883	0.395874	0.148228	C	2.836004	0.365944	0.231082
C	3.821234	-0.618586	-0.151258	C	3.804477	-0.629395	-0.172086
S	1.554862	-1.809636	-0.174091	S	1.478931	-1.894329	-0.003863
C	5.199496	-0.178899	-0.188888	C	5.135664	-0.171865	-0.337590
C	4.474076	1.953982	0.321824	C	4.487759	1.953883	0.282657
H	4.783979	2.980169	0.509798	H	4.752279	2.991816	0.468736
N	5.498497	1.096949	0.047077	N	5.462123	1.148417	-0.075703
N	3.185251	1.707471	0.386854	N	3.160401	1.647757	0.465629
N	6.204527	-1.033996	-0.457363	N	6.168138	-0.963438	-0.728769
H	7.152028	-0.685620	-0.485614	H	7.044133	-0.511871	-0.955808
H	6.045619	-2.007125	-0.663963	H	6.003198	-1.872814	-1.136141
C	0.293570	0.665720	0.415553	C	0.302465	0.617912	0.613669
H	0.584324	1.656298	0.781298	H	0.594257	1.573992	1.063972
C	-0.661001	0.900114	-0.807719	C	-0.593314	0.977566	-0.638817
H	-0.227213	0.536772	-1.743733	H	-0.115776	0.691900	-1.580150
C	-1.890948	0.097201	-0.376060	C	-1.844714	0.146801	-0.338147
H	-1.736010	-0.958196	-0.632041	H	-1.671818	-0.882400	-0.676479
C	-1.866043	0.268060	1.138806	C	-1.901085	0.188608	1.184089
H	-2.123154	1.306070	1.394143	H	-2.170758	1.202066	1.513522
O	-0.923189	2.295028	-0.882172	O	-0.836749	2.373079	-0.594713
H	-1.538817	2.455627	-1.612605	H	-1.326354	2.630315	-1.389750
O	-3.138669	0.548093	-0.938787	O	-3.062256	0.646557	-0.923639
O	-0.507030	-0.010215	1.440799	O	-0.559345	-0.119583	1.537983
C	-2.865481	-0.664023	1.802006	C	-2.932676	-0.793823	1.712435
H	-2.963228	-0.500847	2.874753	H	-3.086683	-0.720955	2.788465
H	-2.640637	-1.716498	1.605647	H	-2.694973	-1.826544	1.440856
C	3.261507	-1.865346	-0.350709	C	3.223503	-1.882328	-0.325622
H	3.739985	-2.809346	-0.573126	H	3.698746	-2.818328	-0.588731
O	-4.172277	-0.321495	1.237565	O	-4.208605	-0.402479	1.111197
P	-4.427107	-0.255159	-0.354315	P	-4.377938	-0.202829	-0.480944
O	-5.574288	0.853916	-0.454432	O	-5.523307	0.910385	-0.546891
H	-6.350880	0.540542	-0.945370	H	-6.262082	0.649259	-1.119912
O	-4.721498	-1.551615	-1.021067	O	-4.628593	-1.437940	-1.270507

Syn - c-thAMP – B3LYP/6-31+G(d,p) – IEFPCM Water

Ground state / **Excited state**

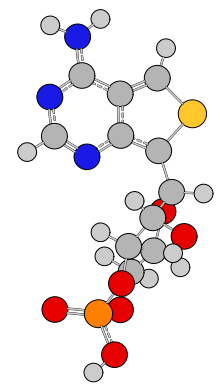

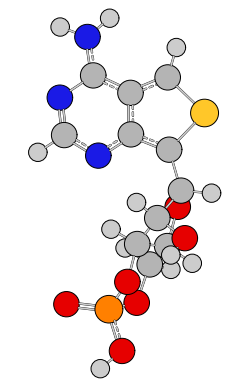

Cartesian coordinates (Ground state)				Cartesian coordinates (Excited state)			
**Atom**	**X**	**Y**	**Z**	**Atom**	**X**	**Y**	**Z**
C	1.709005	1.128132	-0.031063	C	-1.714427	-1.160731	-0.043668
C	2.215495	-0.166649	-0.095662	C	-2.215060	0.169018	-0.101194
C	3.649959	-0.207677	0.057380	C	-3.650885	0.232169	0.057460
S	3.000056	2.257527	0.224699	S	-3.007279	-2.327251	0.232352
C	4.260633	-1.517725	-0.000695	C	-4.227572	1.528615	0.004809
C	2.160344	-2.421003	-0.325906	C	-2.141746	2.451608	-0.329130
H	1.597878	-3.339597	-0.481813	H	-1.525966	3.333706	-0.484725
N	3.503963	-2.597606	-0.191444	N	-3.434508	2.643424	-0.202450
N	1.466067	-1.305019	-0.290510	N	-1.456597	1.258503	-0.294353
N	5.590474	-1.684717	0.130104	N	-5.553934	1.770683	0.132690
H	5.971581	-2.619636	0.103862	H	-5.869939	2.730204	0.162902
H	6.217822	-0.917809	0.313051	H	-6.208336	1.048658	0.394676
C	0.292377	1.593990	-0.094784	C	-0.302219	-1.608321	-0.109352
H	0.262826	2.678202	-0.249343	H	-0.256788	-2.691305	-0.265311
C	-0.600159	0.964586	-1.214905	C	0.591571	-0.961419	-1.223301
H	-0.024886	0.336608	-1.898609	H	0.016259	-0.325814	-1.899692
C	-1.591970	0.156674	-0.376967	C	1.582279	-0.158631	-0.376365
H	-1.124582	-0.802574	-0.129696	H	1.112325	0.797720	-0.123650
C	-1.729169	1.012807	0.876161	C	1.720637	-1.022044	0.871043
H	-2.287942	1.929374	0.635555	H	2.278803	-1.937401	0.624537
O	-1.221999	2.039565	-1.911992	O	1.212278	-2.028608	-1.929741
H	-1.805580	1.669436	-2.590577	H	1.760227	-1.654298	-2.635119
O	-2.876370	-0.064357	-1.000695	O	2.866134	0.069660	-0.997685
O	-0.375132	1.305475	1.185792	O	0.366843	-1.318724	1.182169
C	-2.474035	0.255259	1.962228	C	2.465222	-0.270037	1.961676
H	-2.691595	0.860329	2.841918	H	2.684160	-0.881338	2.836651
H	-1.949119	-0.657344	2.259641	H	1.938334	0.639188	2.265737
C	4.208713	1.044699	0.237850	C	-4.212918	-1.023565	0.236911
H	5.244836	1.325677	0.366859	H	-5.253830	-1.286606	0.370476
O	-3.780567	-0.086708	1.395747	O	3.771034	0.078874	1.397598
P	-3.926861	-0.832059	-0.028385	P	3.913973	0.835692	-0.020947
O	-5.327913	-0.264234	-0.551603	O	5.317282	0.277857	-0.548556
H	-5.952781	-0.971123	-0.779184	H	5.938352	0.989073	-0.773171
O	-3.803908	-2.314526	0.000260	O	3.783834	2.317268	0.018553

Anti - c-^{tz}AMP – B3LYP/6-31+G(d,p) – IEFPCM Water

Ground state

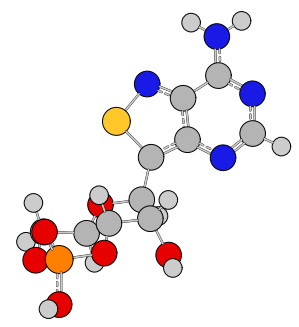

Excited state

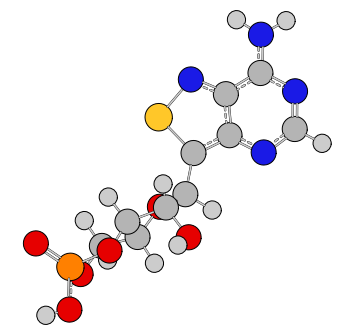

	Cartesian coordinates (Ground state)				Cartesian coordinates (Excited state)		
**Atom**	**X**	**Y**	**Z**	**Atom**	**X**	**Y**	**Z**
C	1.507991	-0.211834	0.569465	C	-1.528029	-0.067460	-0.187368
C	2.710728	0.410035	0.238658	C	-2.856227	0.419136	-0.188603
C	3.642418	-0.565338	-0.236789	C	-3.791362	-0.627057	0.132466
S	1.671441	-1.896253	0.272046	S	-1.583925	-1.794179	0.121212
C	4.947920	-0.076369	-0.630807	C	-5.162090	-0.246045	0.200943
C	4.233933	2.048388	-0.057035	C	-4.599386	1.916717	-0.342061
H	4.518271	3.096695	0.001353	H	-4.909031	2.934598	-0.559418
N	5.211178	1.224210	-0.530898	N	-5.547636	1.053385	-0.038263
N	3.012143	1.747655	0.332008	N	-3.249337	1.691674	-0.376862
N	5.879077	-0.925514	-1.090985	N	-6.134541	-1.130211	0.462723
H	6.786577	-0.578874	-1.366859	H	-7.100966	-0.829689	0.468479
H	5.682193	-1.912304	-1.168203	H	-5.920705	-2.110374	0.594169
C	0.261689	0.412955	1.109128	C	-0.288597	0.709072	-0.376617
H	0.513765	1.072330	1.948323	H	-0.556842	1.731826	-0.662474
C	-0.555186	1.265358	0.081162	C	0.668046	0.829563	0.871678
H	0.020714	1.480778	-0.823117	H	0.232632	0.376523	1.767028
C	-1.734980	0.324352	-0.172055	C	1.900484	0.069395	0.371706
H	-1.426648	-0.445700	-0.889878	H	1.743191	-1.003366	0.538953
C	-1.942568	-0.297155	1.204597	C	1.879531	0.365648	-1.123248
H	-2.348935	0.458490	1.890336	H	2.134843	1.422002	-1.290874
O	-0.949209	2.462119	0.734559	O	0.919741	2.210774	1.075234
H	-1.499822	2.979466	0.128216	H	1.470321	2.314004	1.865360
O	-2.933271	0.968896	-0.641304	O	3.147758	0.469552	0.972976
O	-0.615361	-0.651666	1.576292	O	0.522101	0.110213	-1.453210
C	-2.911474	-1.465114	1.127414	C	2.882861	-0.506563	-1.859064
H	-3.173563	-1.874259	2.102486	H	2.983529	-0.252643	-2.913725
H	-2.545410	-2.263795	0.475686	H	2.657732	-1.572039	-1.753817
O	-4.156254	-0.914324	0.590248	O	4.188577	-0.213836	-1.264309
P	-4.194866	-0.051780	-0.773486	P	4.436333	-0.286442	0.328616
O	-5.428040	0.928651	-0.506627	O	5.587696	0.804540	0.528993
H	-6.102802	0.878874	-1.202640	H	6.358017	0.448715	1.000576
O	-4.246281	-0.834489	-2.036610	O	4.721478	-1.637077	0.882433
N	3.243331	-1.834940	-0.278846	N	-3.300644	-1.841107	0.401145

Syn - c-^{tz}AMP – B3LYP/6-31+G(d,p) – IEFPCM Water

Ground state

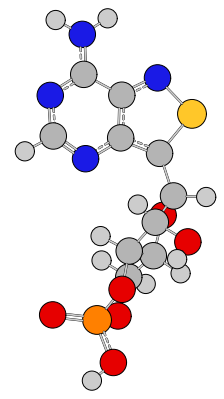

Cartesian coordinates

Atom	X	Y	Z
C	-1.693309	-1.118333	-0.002442
C	-2.179347	0.188692	-0.083406
C	-3.604030	0.183834	0.045705
S	-3.035021	-2.167090	0.226374
C	-4.269957	1.468323	-0.021837
C	-2.190267	2.435261	-0.312327
H	-1.656383	3.371592	-0.458941
N	-3.541241	2.567886	-0.199244
N	-1.456738	1.341754	-0.267937
N	-5.603596	1.547561	0.093460
H	-6.062031	2.446160	0.046002
H	-6.159137	0.715815	0.228309
C	-0.282990	-1.616974	-0.049294
H	-0.272189	-2.705730	-0.168748
C	0.609067	-1.030236	-1.192682
H	0.033187	-0.434145	-1.904828
C	1.590433	-0.182302	-0.379938
H	1.114560	0.781791	-0.169296
C	1.727980	-0.991340	0.904121
H	2.293785	-1.912081	0.701012
O	1.238600	-2.128831	-1.839133
H	1.823560	-1.786067	-2.530923
O	2.874373	0.023956	-1.005005
O	0.372963	-1.285892	1.217437
C	2.459540	-0.188175	1.966053
H	2.675983	-0.758756	2.868664
H	1.926399	0.731010	2.225962
O	3.766010	0.140247	1.393037
P	3.915679	0.835153	-0.056207
O	5.321944	0.257379	-0.551901
H	5.944742	0.958789	-0.801325
O	3.780391	2.316212	-0.082583
N	-4.209036	-0.991388	0.214676

Excited state

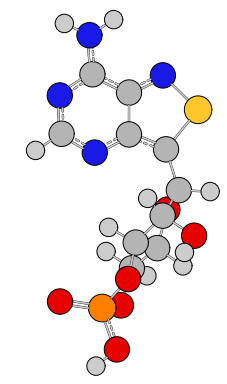

Cartesian coordinates

Atom	X	Y	Z
C	-1.695164	-1.153578	0.053238
C	-2.158085	0.157741	-0.002795
C	-3.603956	0.214858	0.061777
S	-3.109105	-2.213553	0.172701
C	-4.184860	1.514752	-0.051297
C	-2.084505	2.503475	-0.219415
H	-1.472702	3.387392	-0.349139
N	-3.403188	2.621092	-0.137062
N	-1.479707	1.322319	-0.024892
N	-5.530148	1.672566	-0.114929
H	-5.901061	2.564529	-0.414668
H	-6.103939	0.853931	-0.267799
C	-0.301337	-1.656752	0.020025
H	-0.297228	-2.749934	-0.050793
C	0.603046	-1.128827	-1.146394
H	0.025586	-0.583922	-1.898322
C	1.572265	-0.229111	-0.378059
H	1.084857	0.736065	-0.192015
C	1.733245	-0.985474	0.936149
H	2.310251	-1.904896	0.759844
O	1.250936	-2.252809	-1.727242
H	1.842976	-1.940683	-2.427704
O	2.842720	-0.015920	-1.024633
O	0.387831	-1.284122	1.274939
C	2.467068	-0.132096	1.956804
H	2.703692	-0.665670	2.876763
H	1.923582	0.788140	2.190532
O	3.762200	0.195267	1.356043
P	3.882190	0.844611	-0.115967
O	5.291719	0.275437	-0.611452
H	5.898415	0.978506	-0.893917
O	3.717711	2.320957	-0.190935
N	-4.245934	-0.938288	0.226819

7.2 Frontier Molecular Orbitals

The molecular orbitals involved in the S_0–S_1 transition are listed below (Figures S19 and S20). These MOs were obtained from the previously optimized geometries using B3LYP/6-31+G(d,p). Each transition was associated with its configuration interaction coefficient, thus allowing us to determine its proportion.

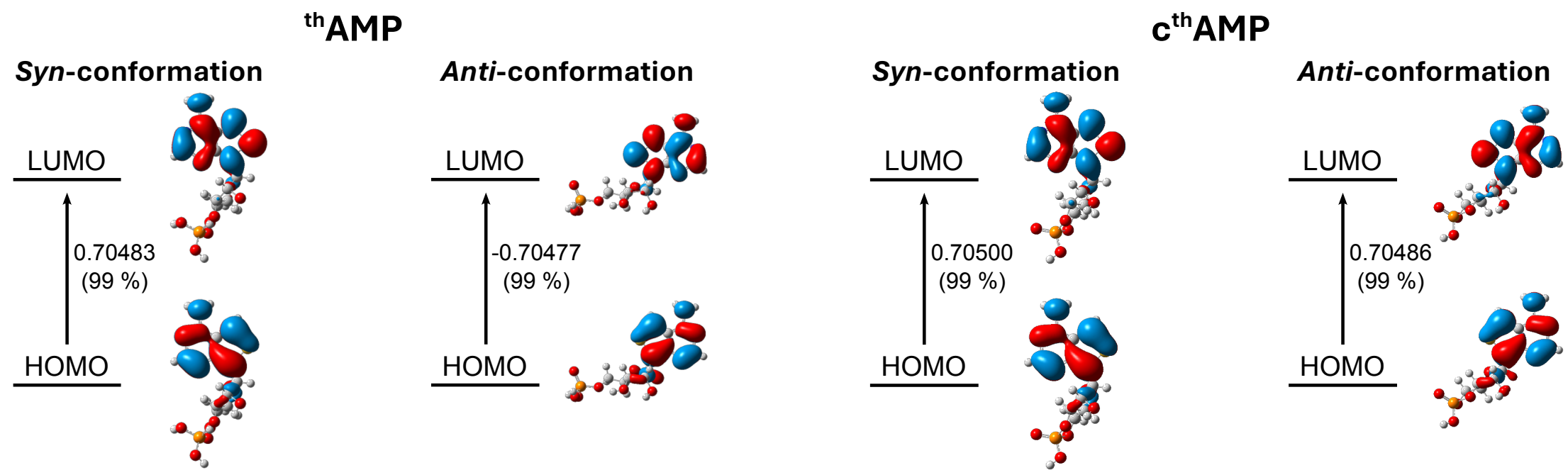

Figure S19. Molecular orbitals involved in the S_0–S_1 transition of thAMP (left) and cthAMP (right) associated with their configuration interaction coefficients.

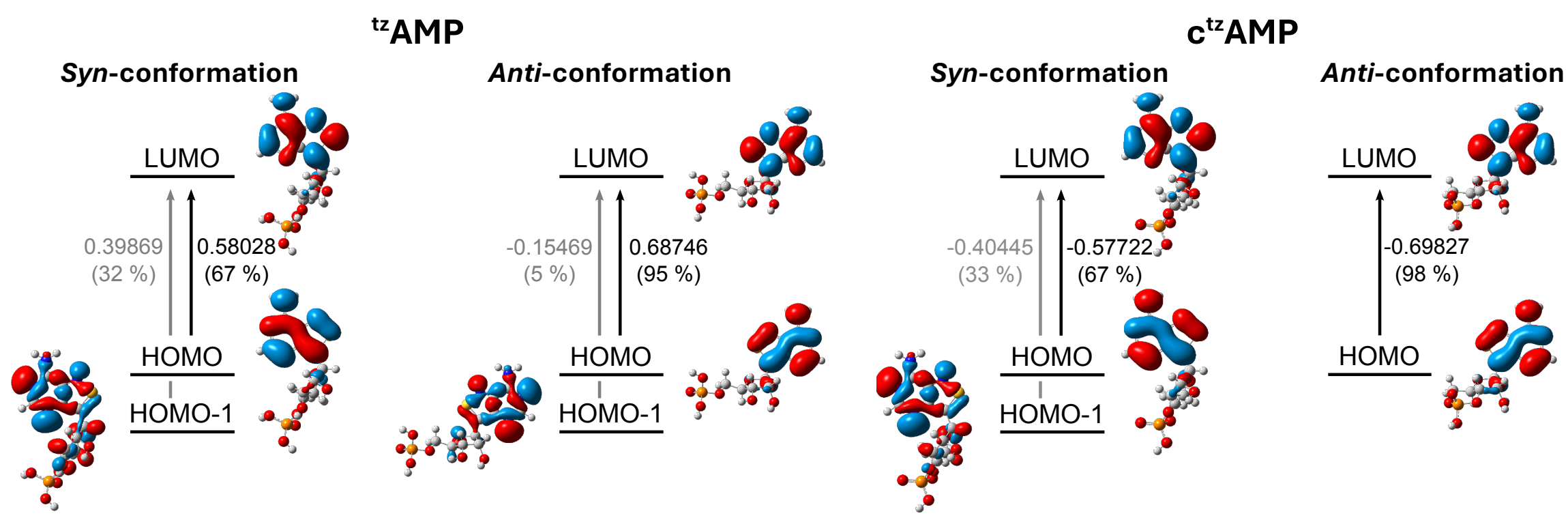

Figure S20. Molecular orbitals involved in the S_0–S_1 transition of ^{tz}AMP (left) and c^{tz}AMP (right) associated with their configuration interaction coefficients.

7.3 Vertical Transition Energies

The vertical transition energies (excitation, emission, and TICT) were computed using B3LYP/6-31+G(d,p) – IEFPCM water and were calculated in **6** steps as described below (Figure S21).[8]

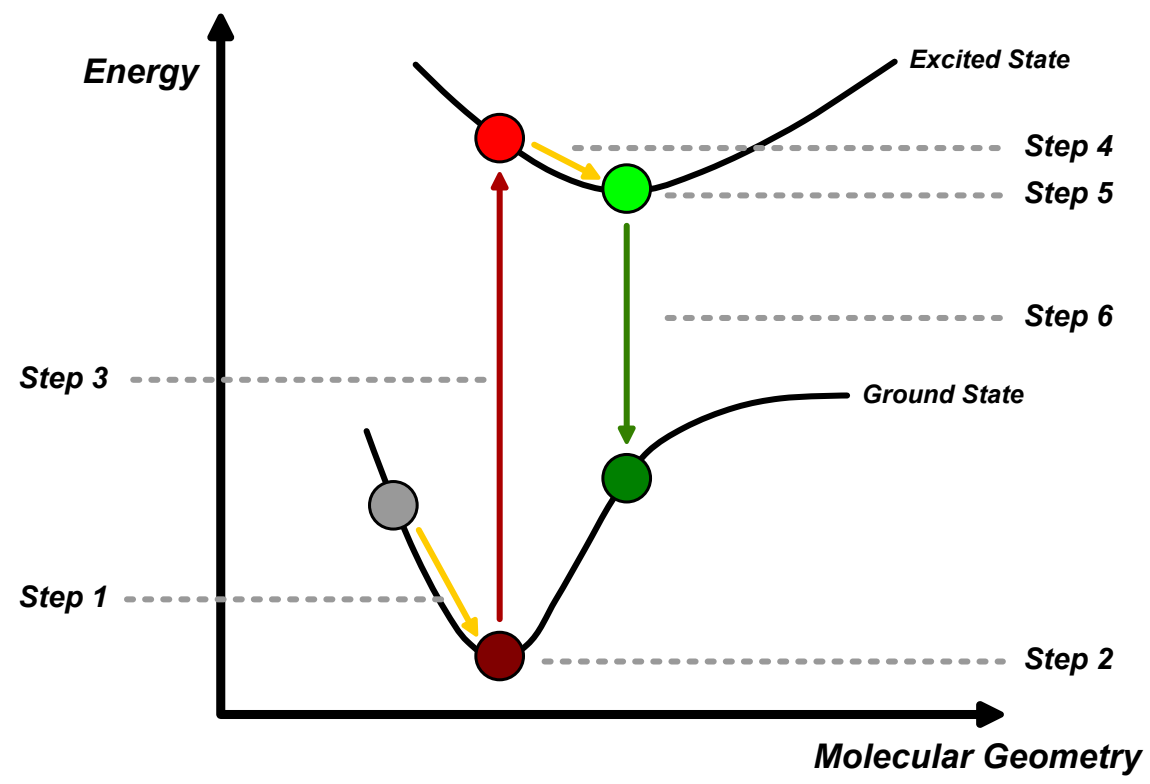

Figure S21. Scheme of the steps involved in the determination of vertical energy transitions (absorption and emission)

Step 1: Geometry optimization of the ground state S_0.

Step 2: From the optimized geometry of step 1, → single point energy of the ground state S_0 using the keyword "noneq=write" to lock the solvent in its "ground state" conformation.

Step 3: Single point energy of the excited state S_1 using the keyword "noneq=read" to consider the solvent in its "ground state" conformation. The energy difference between steps 2 and 3 yields the Franck Condon vertical energy transition.

Step 4: Geometry optimization of the excited state S_1.

Step 5: From the optimized geometry of step 4, → single point energy of the excited state S_1 using the keyword "noneq=write" to lock the solvent in its "excited state" conformation.

Step 6: Single point energy of the ground state S_0 using the keyword "noneq=read" to consider the solvent in its "excited state" conformation. The energy difference between steps 5 and 6 yields the local excited-state vertical energy transition.

7.4 Potential Energy Surfaces in Water

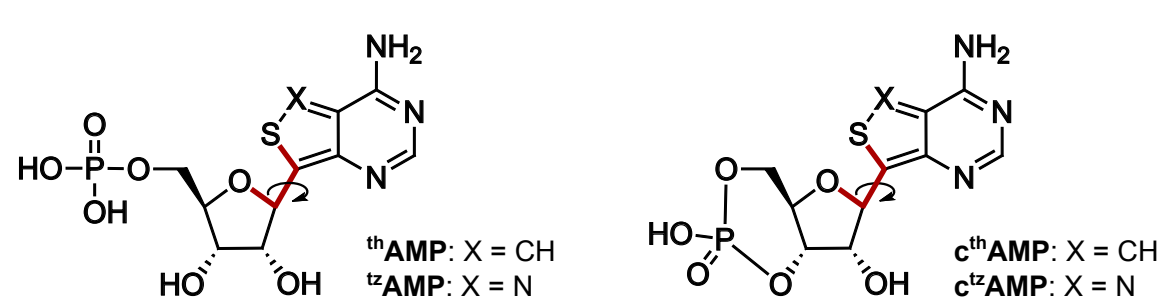

Figure S22. Dihedral angle scanned for **thAMP**, **tzAMP**, **cthAMP**, and **ctzAMP**.

Potential energy surfaces (PES) were computed using B3LYP 6-31+G(d,p). As for the optimized geometries, IEFPCM was employed to account for the solvent (water). The dihedral angles around the glycosidic bond (S–C^9–$C^{1'}$–O, Figure S22) were scanned from 0° to 360° (steps of 15°) starting from the previously optimized geometries. The dihedral angles were fixed, while other geometry parameters were freely optimized at each step (relaxed scan).

References

[1] F. Hallé, A. Fin, A. R. Rovira, Y. Tor, Emissive Synthetic Cofactors: Enzymatic Interconversions of tzA Analogues of ATP, NAD+, NADH, NADP+, and NADPH. *Angewandte Chemie International Edition* **57**, (2018): 1087–1090.

[2] J. Feldmann, Y. Li, Y. Tor, Emissive Synthetic Cofactors: A Highly Responsive NAD+ Analogue Reveals Biomolecular Recognition Features. *Chemistry – A European Journal* **25**, (2019): 4379–4389.

[3] R. K. Harris, E. D. Becker, S. M. C. De Menezes, P. Granger, R. E. Hoffman, K. W. Zilm, Further Conventions for NMR Shielding and Chemical Shifts (IUPAC Recommendations 2008) International Union of Pure and Applied Chemistry Physical and Biophysical Chemistry Division. *Magnetic Resonance in Chemistry* **46**, (2008): 582–598.

[4] S. Strickler, R. A. Berg, Relationship between absorption intensity and fluorescence lifetime of molecules. *The Journal of Chemical Physics* **37**, (1962): 814.

[5] B. Cohen, C. E. Crespo-Hernández, B. Kohler, Strickler–Berg analysis of excited singlet state dynamics in DNA and RNA nucleosides. *Faraday Discussions* **127**, (2004): 137–147.

[6] R. F. Ribeiro, A. V. Marenich, C. J. Cramer, D. G. Truhlar, Use of solution-phase vibrational frequencies in continuum models for the free energy of solvation. *The Journal of Physical Chemistry B* **115**, (2011): 14556–14562.

[7] G. Luchini, J. V. Alegre-Requena, I. Funes-Ardoiz, R. S. Paton, GoodVibes: automated thermochemistry for heterogeneous computational chemistry data. *F1000Research* **9**, (2020): 291.

[8] M. Caricato, B. Mennucci, J. Tomasi, F. Ingrosso, R. Cammi, S. Corni, et al., Formation and relaxation of excited states in solution: A new time dependent polarizable continuum model based on time dependent density functional theory. *The Journal of Chemical Physics* **124**, (2006).
